# Global Regulatory Pathways Converge To Control Expression of Pseudomonas aeruginosa Type IV Pili

**DOI:** 10.1128/mbio.03696-21

**Published:** 2022-01-25

**Authors:** Kimberly A. Coggan, Matthew G. Higgs, Evan D. Brutinel, Jeremiah N. Marden, Peter J. Intile, Hanne C. Winther-Larsen, Michael Koomey, Timothy L. Yahr, Matthew C. Wolfgang

**Affiliations:** a Department of Microbiology and Immunology, University of North Carolina, Chapel Hill, North Carolina, USA; b Department of Microbiology and Immunology, University of Iowa, Iowa City, Iowa, USA; c Marsico Lung Institute, University of North Carolina, Chapel Hill, North Carolina, USA; d Center for Integrative Microbial Evolution and Department of Pharmacology and Pharmaceutical Biosciences, University of Oslo, Oslo, Norway; e Department of Biosciences, Section for Genetics and Evolutionary Biology and Centre for Ecological and Evolutionary Synthesis, University of Oslo, Oslo, Norway; University of Washington

**Keywords:** AlgR, *Pseudomonas aeruginosa*, Vfr, cAMP, type IV pili

## Abstract

The opportunistic pathogen Pseudomonas aeruginosa relies upon type IV pili (Tfp) for host colonization and virulence. Tfp are retractile surface appendages that promote adherence to host tissue and mediate twitching motility, a form of surface-associated translocation. Tfp are composed of a major structural pilin protein (PilA), several less abundant, fiber-associated pilin-like proteins (FimU, PilV, PilW, PilX, and PilE), and a pilus-associated tip adhesin and surface sensor (PilY1). Several proteins critical for Tfp biogenesis and surface sensing are encoded by the *fimU-pilVWXY1Y2E* operon. Tfp biogenesis is regulated by the global transcription factor Vfr and its allosteric effector, cyclic AMP (cAMP). Our investigation into the basis for reduced Tfp production in cAMP/*vfr* mutants revealed a defect in the expression of the *fimU* operon. We found that cAMP/Vfr activation of the *fimU* operon occurs via direct binding of Vfr to a specific *fimU* promoter sequence. We also refined the role of the AlgZ/AlgR two-component system in *fimU* regulation by demonstrating that phosphorylation of the response regulator AlgR is required for maximal binding to the *fimU* promoter region *in vitro*. Vfr also regulates expression of the *algZR* operon, revealing an indirect regulatory loop affecting *fimU* operon transcription. Overall, these results demonstrate that two linked but independent regulatory systems couple the expression of Tfp biogenesis and surface sensing genes and highlight the regulatory complexity governing expression of P. aeruginosa virulence factors.

## INTRODUCTION

Pseudomonas aeruginosa is an opportunistic pathogen that causes a wide variety of diseases ranging from acute and disseminating tissue infections to debilitating chronic lung infections in individuals with muco-obstructive lung disease (1–3). Acute P. aeruginosa infections are characterized by an intense neutrophil-dominated inflammatory response, extensive tissue damage, and sepsis. In contrast, chronic infections of the cystic fibrosis (CF) lung are noninvasive and cause progressive damage and respiratory decline over time. P. aeruginosa virulence relies on the expression of many surface-exposed and secreted factors (4, 5), and individual virulence factors are largely associated with either the acute or chronic mode of infection (6). Acute infection is partially mediated by direct injection of exotoxins into host cells by a type III secretion system (T3SS) and a variety of toxins, degradative proteases, and lipases secreted by a type II secretion system (T2SS) (7). Acute virulence is also mediated by type IV pili (Tfp), which are filamentous surface appendages that promote interactions with host cells (8). Chronic infection is typified by a biofilm-like mode of growth, where bacteria exist as microcolonies encased in one of several exopolysaccharides (9). Chronic CF isolates commonly exhibit a mucoid colony phenotype, resulting from overproduction of the exopolysaccharide alginate (10).

Despite the association of specific P. aeruginosa factors with either acute or chronic infection lifestyles, Tfp have the potential to contribute to both infection modes. In addition to host cell attachment (8), Tfp also mediate a form of surface-associated motility (twitching motility) that promotes bacterial dissemination within infected tissue (11). Tfp are also important for the initiation of biofilm formation (12) and play a role in maintaining biofilm structure (13). The various functions of Tfp fibers are mediated by rapid, repeated cycles of extension and retraction (14). The Tfp fiber is primarily composed of pilin (or PilA) subunits, which are produced as prepilins containing a short amino-terminal leader sequence that is cleaved prior to assembly (15). Assembly and disassembly of Tfp occur in an energy-dependent manner at the inner membrane via the ATPases PilB and PilT, which respectively facilitate extension and retraction of the pilus fiber through an outer membrane pore (16). In addition to pilin, several less abundant minor pilins (FimU, PilV, PilW, PilX, and PilE), are required for pilus assembly, function, and surface stability (17–22). The minor pilins are present in the mature pilus fiber (23) and believed to form a complex at regular intervals along the length of the fiber or at the fiber tip. PilY1 is a multifunctional pilus-associated protein that antagonizes pilus retraction (22) and acts as an adhesin for abiotic surfaces and integrins located at the basolateral membrane of human epithelial cells (8, 24, 25). PilY1 also functions as a mechanosensor, linking attachment with surface-associated virulence behaviors and fiber retraction (25–28). Upon contact with a surface, PilY1 regulates virulence by modulating the production of alkyl quinolones through regulation of the AlgZR two-component system (29, 30). In addition, PilY1 sensing is associated with repression of swarming motility and implicated in other surface-associated behaviors, such as biofilm formation, through the regulation of cyclic di-GMP (26, 31, 32).

Multiple P. aeruginosa signal transduction pathways participate in the reciprocal regulation of virulence factors associated with acute and chronic infection (6). The AlgZR two-component system (TCS) regulates multiple virulence systems. AlgZ (also referred to as FimS) and AlgR are both required for twitching motility and Tfp production, suggesting that AlgR phosphorylation by AlgZ is required for activity (33–35). AlgR controls transcription of the *fimU-pilVWXY1Y2E* operon (*fimU*) (17–20, 35). Proteins encoded within the *fimU* operon negatively regulate AlgR activity through a poorly understood mechanism that appears to involve surface sensing by PilY1 (27, 28, 30, 32). AlgR also mediates the conversion to mucoidy (alginate overproduction) that occurs during chronic infection in the CF lung (36–39).

The global transcriptional regulator Vfr coordinately controls the expression of over 200 genes, many of which are involved in P. aeruginosa virulence (40–42). Vfr activity is regulated by the second messenger cyclic AMP (cAMP), which is synthesized by two adenylate cyclases (CyaA and CyaB) (42–45). Vfr was identified in a screen for twitching motility-defective mutants (43), and Tfp production requires both Vfr and cAMP (41, 46). Furthermore, spontaneous *mucA* mutation, a common adaptation found in chronic-CF isolates, results in downregulation of invasive virulence factors via inhibition of *vfr* expression, an effect that involves both AlgU and AlgR (41). Thus, when overexpressed due to *mucA* mutation, AlgR not only mediates the switch to a chronic infection phenotype via activation of alginate production but also represses acute virulence factors through inhibition of cAMP/Vfr signaling.

Although cAMP/Vfr signaling is implicated in Tfp biogenesis and function, the exact mechanism of this regulation is unknown. In this study, we sought to identify the specific defect in cAMP/Vfr signaling mutants that accounts for loss of Tfp biogenesis and determine how the cAMP/Vfr system is integrated within the complex Tfp biogenesis regulatory network.

## RESULTS

### Vfr/cAMP and AlgZR control surface abundance of Tfp.

**FIG 1:**
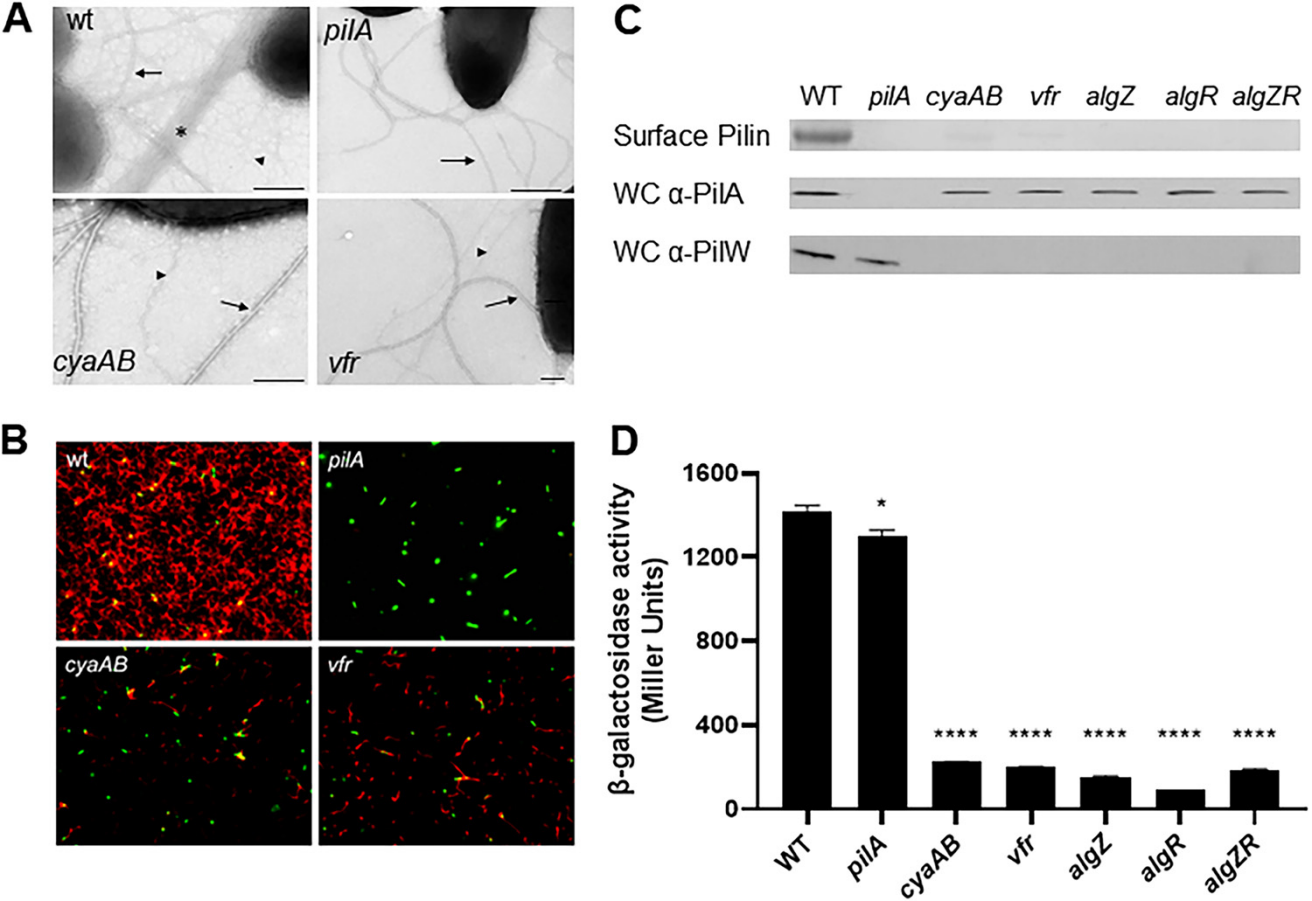
P. aeruginosa adenylate cyclases and Vfr are required for Tfp production. (A) Visualization of Tfp (thin fibers and fiber bundles) produced by wild-type (wt) and mutant P. aeruginosa strains by TEM. Thin Tfp fibers and fiber bundles are indicated by arrowheads and an asterisk, respectively. Flagella are indicated by arrows. Bar = 250 nm. (B) Tfp abundance on wild-type and mutant P. aeruginosa strains by IF microscopy. P. aeruginosa cells (green) were identified with Hoechst stain, and Tfp (red) were detected with pilin-specific antiserum and Alexa Red-conjugated goat anti-rabbit IgG (Molecular Probes). Representative IF micrographs are shown at a magnification of ×100. (C) (Top) Coomassie blue-stained SDS-polyacrylamide gel showing surface pilin from purified pilus fractions isolated from the wild-type (WT) and indicated mutant strains. (Middle and bottom) Immunoblots of whole-cell lysates (WC) probed with pilin-specific monoclonal antibody or PilW-specific antiserum. An uncropped blot showing equal protein loading is shown in Fig. S1A. (D) Activity of the *fimU* promoter as measured by β-galactosidase assay in the indicated strains harboring a chromosomal P*_fimU_*-*lacZ* transcriptional reporter. Values are means and standard errors of the means (SEM) (*n *≥ 4). When compared pairwise, values for all mutants were significantly different (*, *P* < 0.05; ****, *P *< 0.0001) from those for the wild-type strain as determined by one-way analysis of variance (ANOVA) with multiple comparisons using Bonferroni’s correction.

Although both Vfr and cAMP are required for Tfp production and function, a mechanism for this requirement has not been determined (41, 43, 46). To characterize the Tfp defect in strains lacking Vfr or cAMP production, we evaluated the abundance and morphology of Tfp surface fibers by transmission electron microscopy (TEM). TEM images of the wild-type strain show both flagella (thick fibers) and abundant Tfp (thin fibers and fiber bundles) extending from the bacterial surface (Fig. 1A). In contrast, the *vfr* mutant and a *cyaA cyaB* (*cyaAB*) double mutant, which lacks the two adenylate cyclases necessary for cAMP synthesis, showed a substantial reduction in surface Tfp. The length, diameter (relative to flagella), and morphology of surface Tfp produced by the *vfr* and *cyaAB* mutants were indistinguishable from those of Tfp produced by the wild-type strain. Both mutants displayed approximately 1 to 2 Tfp fibers per cell, as assessed by evaluating bacteria in multiple TEM images (data not shown). Despite the dramatic reduction in Tfp, both the *vfr* and *cyaAB* mutants could be distinguished from a nonpiliated *pilA* mutant lacking the major pilus structural subunit (47).

10.1128/mbio.03696-21.1 FIG S1 Uncropped α-PilW Western blots. α-PilW blots for Fig. 1C (A), Fig. 5B and C (B), and Fig. 9B (C). Blots for PilW and PilA were run on the same samples, which were generated as described in Materials and Method. Protein was normalized based on total protein levels as determined by a Bradford assay. Download FIG S1, TIF file, 0.5 MB. Copyright © 2022 Coggan et al. 2022 Coggan et al. https://creativecommons.org/licenses/by/4.0/ This content is distributed under the terms of the Creative Commons Attribution 4.0 International license.

To confirm the TEM results, wild-type and mutant P. aeruginosa strains were labeled with pilin-specific antibodies and imaged by immunofluorescence (IF) microscopy (Fig. 1B). This technique allows the direct visualization of Tfp abundance on the surface of live bacteria (8, 48). When examined by IF, the wild-type strain displayed an extensive network of Tfp fibers, and the *pilA* mutant lacked any appreciable Tfp staining. The *cyaAB* and *vfr* mutants both displayed considerably less Tfp staining than the wild type, indicating that the reduction in Tfp seen by TEM was not due to Tfp shedding but rather reflects a reduction in Tfp expression and/or elaboration on the surface.

As pilin is the major structural component of Tfp, we also evaluated the relative amounts of pilin recovered in sheared pilus preparations, which correlates with surface Tfp abundance. As previously demonstrated (41, 43, 46), the *vfr* and *cyaAB* mutants displayed dramatically reduced levels of surface pilin compared to the wild-type strain (Fig. 1C, top). With the exception of the *pilA* mutant, similar levels of pilin were detected in whole-cell lysates of all strains by immunoblotting (Fig. 1C, middle), confirming that the reduction in surface Tfp seen in the *vfr* and *cyaAB* mutants is due to a defect in fiber assembly rather than a consequence of reduced pilin synthesis.

The AlgZR two-component system controls Tfp production by directly regulating the *fimU* operon (35, 49). To determine whether the AlgZR and cAMP/Vfr pathways play a similar regulatory role in Tfp production, we assessed the relative abundance of surface and total cellular pilin in mutants with disruption of either *algZ* and *algR* individually or in combination (*algZR*). All three mutants lacked detectable surface Tfp (Fig. 1C, top) but retained the capacity to synthesize pilin (Fig. 1C, middle). Next, we assessed the impact of *vfr*, *cyaAB*, *algZ*, and *algR* mutations on *fimU* operon expression. As a readout for *fimU* operon expression, we initially evaluated the level of PilW, a protein encoded within the *fimU* operon. Each of the mutants produced substantially less PilW than the wild-type strain, as determined by immunoblotting of whole-cell lysates (Fig. 1C, bottom). Raw immunoblotting data, demonstrating equal sample loading, are available in Fig. S1 in the supplemental material.

As an alternative readout for the presence of functional pilin, we performed a phage susceptibility assay using the pilus-specific lytic phage PO4 (50). PO4 infection requires both surface-accessible pili and pilus retraction for attachment and entry, respectively (16). Despite possessing dramatically reduced surface pili, the *vfr*, *cyaAB*, and *algZR* mutants were indistinguishable from the wild type and exhibited complete sensitivity to PO4 (Fig. S2). In contrast, an isogenic *pilA* mutant was resistant to lysis even at the highest phage concentration. This result suggests that, despite the lack of appreciable surface fibers, the *vfr*, *cyaAB*, and *algZR* mutants can assemble pilus structures that are sufficient to support phage attachment and entry. Given that proteins encoded in the *fimU* operon are required for pilus stability (22), we hypothesize that the regulatory mutants may produce dysfunctional pili that can extend only to the bacterial cell surface or outer membrane prior to retraction, such that they can support phage attachment and entry but do not form conspicuous surface fibers. Regardless of the specific defect, these findings support the notion that cAMP/Vfr and AlgZR both control a functionally related aspect of pilus biogenesis.

10.1128/mbio.03696-21.2 FIG S2 *vfr*, *cyaAB*, and *algZR* mutants exhibit sensitivity to the pilin-specific phage PO4. Phage susceptibility was determined using a double-agar layer as described in Materials and Methods. Phage was serially diluted from 10^8^ PFU/mL to 10^0^ PFU/mL. Ten microliters of each phage dilution, as well as broth control, was spotted onto double agar containing the indicated strains and incubated overnight before imaging. Phage concentrations above 10^5^ PFU/mL also resulted in complete lysis. The assay was performed in duplicate. Download FIG S2, TIF file, 0.9 MB. Copyright © 2022 Coggan et al. 2022 Coggan et al. https://creativecommons.org/licenses/by/4.0/ This content is distributed under the terms of the Creative Commons Attribution 4.0 International license.

To determine if altered transcription of the *fimU* operon accounts for reduced PilW in the cAMP/Vfr and *algZR* mutants, we constructed a transcriptional reporter by fusing the putative *fimU* promoter region (P*_fimU_*) to the β-galactosidase-encoding *lacZ* gene and introduced the construct onto the chromosomes of wild-type and mutant strains. Each of the mutants showed a significant reduction in P*_fimU-_lacZ* reporter activity compared to that of the wild-type strain (Fig. 1D). Wild-type levels of P*_fimU_*-*lacZ* reporter activity were restored in each of the mutants by plasmid-based expression of the corresponding genes in *trans* (Fig. S3). These results demonstrate that cAMP/Vfr and AlgZR are required for optimal expression of the *fimU* operon and confirm previous reports (42, 43).

10.1128/mbio.03696-21.3 FIG S3 Restoration of P*_fimU_*-*lacZ* reporter activity in mutants. The wild-type (WT) or indicated mutant strains containing either vector, pPa-*cyaB*, pPa-*vfr*, pPa-*algZ*, pPa-*algR*, or pPa-*algZR* expression plasmids were grown in LB broth containing 30 μg/mL carbenicillin (Cb) and 50 μM IPTG. Values are means and SEM (*n *≥ 3). Download FIG S3, TIF file, 0.3 MB. Copyright © 2022 Coggan et al. 2022 Coggan et al. https://creativecommons.org/licenses/by/4.0/ This content is distributed under the terms of the Creative Commons Attribution 4.0 International license.

### AlgR affinity for the *fimU* promoter is dramatically enhanced by the phosphodonor phosphoamidate.

**FIG 2:**
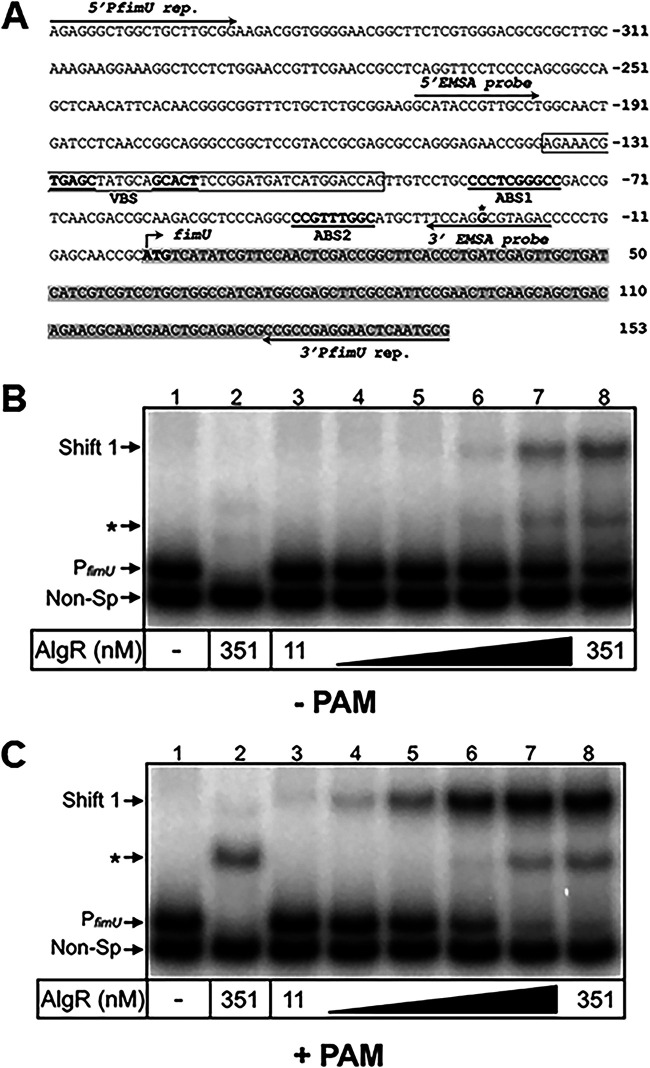
AlgR requires phosphorylation for *fimU* promoter recognition. (A) Diagram of the *fimU* promoter region. Numbering (in base pairs) is relative to the *fimU* translational start site; the ATG is indicated in bold type. The partial coding sequence of *fimU* is highlighted in gray. Locations of oligonucleotides used to generate the P*_fimU_* reporter (5′P*fimU* rep. and 3′P*fimU* rep.) and P*_fimU_* EMSA probes (5′EMSA probe and 3′ EMSA probe) are indicated. The boxed region represents the sequence protected by cAMP-Vfr in DNase I footprinting assays (Fig. S4) with the identified VBS (centered at −130 and −115 bp) is indicated in bold and underlined. The previously identified AlgR binding sites (ABS1 and ABS2) are indicated. (B and C) Specific (P*_fimU_*, lanes 1 and 3 to 8) and nonspecific (Non-Sp, lane 2) probes (0.25 nM) were incubated in the absence (lane 1) or presence (lanes 2 to 8) of various concentrations of AlgR (11 to 351 nM) for 15 min, followed by electrophoresis and phosphorimaging. Reactions were performed in the absence (B) or presence (C) of the small molecule phosphodonor PAM (50 mM). The nonspecific probe shift is indicated by an asterisk.

The *fimU* promoter region contains two putative AlgR binding sites (designated ABS1 and ABS2) (Fig. 2A) (35). Our finding that both *algZ* and *algR* are required for activation of the *fimU* promoter (Fig. 1D) suggested that AlgZ-dependent phosphorylation of AlgR is required for P*_fimU_* activation. This hypothesis is consistent with the finding that an *algZ* mutant is defective for Tfp-dependent function (34). Furthermore, an AlgR mutant lacking the phospho-accepting aspartate residue (AlgRD54N) is defective for Tfp production and unable to bind the *fimU* promoter *in vitro* (34, 35). Despite these findings, direct evidence that phosphorylation increases AlgR DNA binding activity is lacking. To confirm AlgR binding to the *fimU* promoter and to assess the impact of phosphorylation, we purified full-length AlgR with an amino-terminal histidine fusion tag and assessed binding to a P*_fimU_* promoter probe in the presence and absence of small-molecule phosphodonor compounds. In the absence of a phosphodonor, binding to the P*_fimU_* probe was observed only when high concentrations of AlgR (351 nM) were used, and less than 50% of the probe was shifted (Fig. 2B). AlgR bound to a nonspecific probe devoid of identifiable AlgR binding sites with similar kinetics, suggesting that binding to P*_fimU_* in the absence of AlgR phosphorylation is not biologically relevant (Fig. 2B). In contrast, addition of the phosphodonor phosphoamidate (PAM) significantly enhanced AlgR binding to the P*_fimU_* promoter probe (Fig. 2C). We also observed an increase in the intensity of the nonspecific probe shift; however, specific binding was enhanced to a greater degree. Although the exact increase in affinity could not be calculated due to the poor binding activity of unphosphorylated AlgR, we estimate that affinity of AlgR for the P*_fimU_* promoter probe increased by at least 1 order of magnitude in the presence of PAM. These results confirm that AlgR binds the *fimU* promoter and that phosphorylation is required for specific high-affinity binding and support the hypothesis that AlgZ functions as the cognate sensor kinase responsible for AlgR phosphorylation. Although Belete et al. (35) reported two shifted products, our results show a single shift product (Fig. 2C). While two putative AlgR binding sites have been identified (35), our results suggest that only a single site is occupied or that AlgR binding is cooperative such that both sites become occupied simultaneously. Further studies are required to determine the individual contributions of the two putative AlgR binding sites.

10.1128/mbio.03696-21.4 FIG S4 Vfr protected region of the *fimU* operon. DNase I footprinting of the *fimU* promoter region. Samples contained a DNA fragment (0.4 nM) corresponding to ∼−300 bp relative to the translational start site. The top strand of the DNA probe was radiolabeled on one end and was incubated in the absence or presence of cAMP-Vfr (114, 13, or 1 nM) prior to DNase I treatment. DNase I-generated fragments were separated by electrophoresis, and Maxam-Gilbert sequencing ladders were made using the same DNA. Sequence corresponding region protected by Vfr binding is bracketed, with the predicted half-sites underlined and in bold. Download FIG S4, TIF file, 0.6 MB. Copyright © 2022 Coggan et al. 2022 Coggan et al. https://creativecommons.org/licenses/by/4.0/ This content is distributed under the terms of the Creative Commons Attribution 4.0 International license.

### Vfr directly controls *algZ* expression.

**FIG 3:**
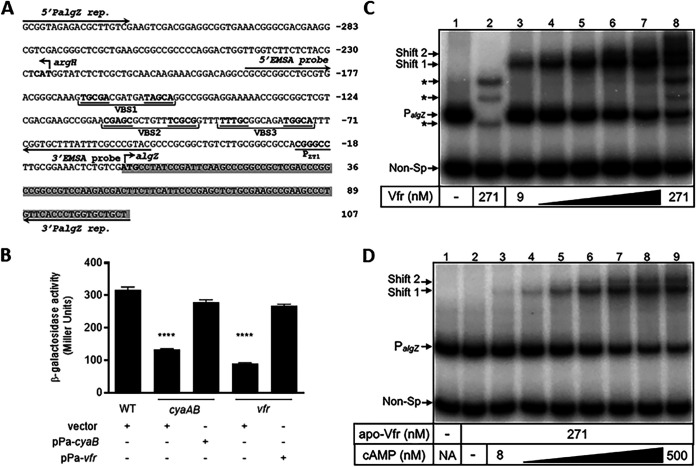
Vfr is required for *algZ* promoter expression. (A) Diagram of the *algZ* promoter region. Numbering (in base pairs) is relative to the *algZ* translational start site. Also depicted is the divergently transcribed gene *argH*. The translational start codons are indicated in bold type. Partial coding sequences of *algZ* and *argH* are highlighted in gray. The proposed Vfr-binding sites VBS1 (centered between −166 and −151 bp), VBS2 (centered between -109 and −94 bp), and VBS3 (centered between −89 and −74) are indicated in bold and underlined. Locations of the oligonucleotides (5′P*algZ* rep. and 3′P*algZ* rep.) used to generate the P*_algZ_*-*lacZ* reporter fragment and P*_algZ_* EMSA probe used for the lower panel are indicated. The Vfr dependent P_ZT1_ is located 23 bp upstream of *algZ*, whereas the Vfr-independent P_ZT2_ (not shown) is located 1,044 bp upstream of *algZ*. (B) Activity of the *algZ* promoter as measured by β-galactosidase assay in the indicated strains harboring a chromosomal P*_algZ_*-*lacZ* transcriptional reporter. The wild-type (WT) or indicated mutant strains contained vector, pPa-*cyaB*, or pPa-*vfr* expression plasmids. Strains containing expression vectors were grown in LB broth containing 30 μg/mL carbenicillin (Cb) and 50 μM IPTG. Values are means and SEM (*n *≥ 3). Values for the *cyaAB* and *vfr* mutants containing empty vector were significantly different from that of WT (*P* ≤ 0.0001), as determined by one-way ANOVA with multiple comparisons using Bonferroni’s correction. (C) Specific probe (lanes 1 and 3 to 8) and nonspecific probe (lane 2) (0.25 nM) were incubated in the absence (lane 1) or presence (lanes 2 to 8) of various concentrations of cAMP-Vfr (9 to 271 nM) for 15 min followed by electrophoresis and phosphorimaging. Bands representing shifting of the nonspecific probe (∼200 bp) are indicated by an asterisk. Arrows indicate the two cAMP-Vfr-dependent shift complexes (Shift 1 and Shift 2). (D) Apo-Vfr (271 nM) incubated with specific (P*_algZ_*, lanes 1 and 3 to 8) and nonspecific (Non-Sp, lane 2) probes (0.25 nM) in the absence (lane 2) or presence (lanes 3 to 9) of increasing concentrations of cAMP (8 to 500 nM) for 15 min followed by electrophoresis and phosphorimaging.

Based on the evidence that AlgZR positively regulates the *fimU* operon and that *algZ* expression was reduced in a *vfr* mutant (42), we hypothesized that decreased *algZR* expression in the cAMP and *vfr* mutants could account for the defect in Tfp production observed. AlgZR are coexpressed from two promoters (designated ZT1 and ZT2). Expression of the proximal ZT1 promoter (Fig. 3A) is entirely Vfr dependent, whereas the far-upstream ZT2 promoter is Vfr independent (51). To examine the role of the Vfr-dependent *algZ* promoter, we engineered an *algZ* transcriptional reporter (P*_algZ_-lacZ*) by fusing the putative Vfr-dependent ZT1 promoter to *lacZ.* P*_algZ-_lacZ* reporter activity was significantly reduced in the *vfr* and *cyaAB* mutants, and the defect was complemented by plasmid-based expression of *vfr* (pPa-*vfr*) or *cyaB* (pPa-*cyaB*), respectively (Fig. 3B).

**FIG 4:**
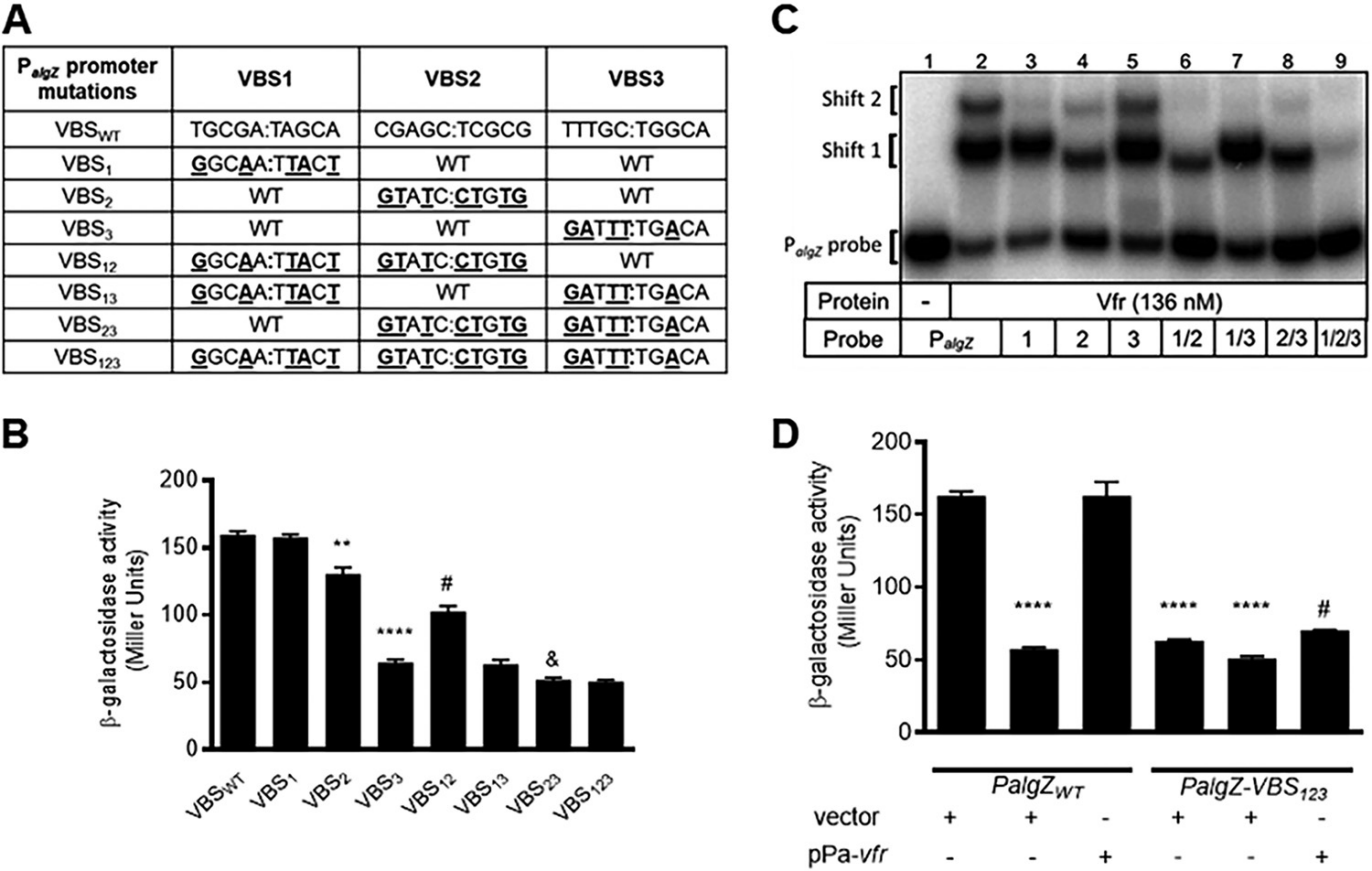
VBS 2 and 3 are required for full activity of Vfr-dependent *algZ* promoter expression. (A) Putative Vfr binding sites (VBS). Mutations in different VBS were generated by site-directed mutagenesis. (B) Promoter activity in strains containing either the wild-type or mutant P*_algZ_-lacZ* reporter as measured by β-galactosidase assay. Values are the means and SEM (*n *≥ 3). Values for VBS2 and VBS3 mutants were significantly different when compared pairwise to that of the wild-type strain (**, *P* < 0.01; ****, *P* < 0.0001). Combinatorial VBS1-2 and VBS2-3 mutations produced additive reporter activity losses compared to individual VBS2 (*#*, *P* < 0.05) and VBS3 (&, *P* < 0.0001) mutations, respectively. Statistical analysis was performed using one-way ANOVA with multiple comparisons using Bonferroni’s correction. (C) EMSA analysis of P*_algZ_* probes containing individual or combinatorial mutations of putative Vfr binding sites using the EMSA probes indicated in Fig. 3A. A double shift of the wild-type promoter probe is significantly abrogated in a triple VBS1-2-3 P*_algZ_* probe. (D) Promoter activity in strains containing either the wild-type or mutant P*_algZ_-*VBS1-2-3 reporter as measured by β-galactosidase assay. Reporter constructs were in either a wild-type (WT) or *vfr* mutant strain background. ****, *P* < 0.0001 compared to WT + vector; *#*, *P* < 0.0001 compared to *vfr* + p*Pa*-*vfr*. Statistical analysis was performed using one-way ANOVA with multiple comparisons using Bonferroni’s correction.

Kanack et al. previously demonstrated that Vfr bound a DNA probe encompassing the *algZR* promoter region by electrophoretic mobility shift assay (EMSA) (52). Based on visual inspection, the authors identified a putative Vfr-binding site (5′-TTT**TTTGC**GGC:AGA**TGGCA**TTT [boldface indicates the conserved half-sites to which Vfr is predicted to bind, and a colon represents the axis of symmetry within the binding site]), although direct binding to this site was not confirmed (52). Based on the Vfr-binding consensus sequence (VCS, 5′-ANWW**TGNGA**WNY:AGW**TCACA**T), we identified two additional candidate Vfr-binding sites, 5′-GAA**CGAGC**GCT:GTT**TCGCG**GTT and 5′-AAG**TGCGA**CGA:TGA**TAGCA**GGC in the *algZ* promoter region (44). We designated these putative binding sites VBS1, VBS2, and VBS3 based on their arrangement within the *algZ* promoter region (Fig. 3A). We first confirmed that purified Vfr binds to a P*_algZ_* promoter probe encompassing all three putative Vfr binding sites by EMSA. Two shift products were produced with increasing concentrations of cAMP-saturated Vfr, consistent with Vfr binding to at least two distinct sites (Fig. 3C, lane 8). The alternative possibility that the second shift is due to Vfr oligomerization at a single site is addressed below (Fig. 4). Vfr binding to an unrelated DNA probe was observed only at the highest Vfr concentrations tested, demonstrating that binding to the *algZ* promoter probe is highly specific (Fig. 3C, lanes 7 and 8). To determine the role of cAMP in Vfr binding, we generated cAMP-free Vfr (apo-Vfr) as previously described (44). Apo-Vfr did not shift the P*_algZ_* probe (Fig. 3D, lane 2), but the two shift products were restored by the addition of cAMP to the binding reactions (Fig. 3D, lanes 3 to 9), demonstrating that Vfr requires cAMP for binding to the P*_algZ_* probe.

To determine which of the putative binding sites contribute to *algZ* transcription and Vfr binding, we disrupted the sites individually and in combination (Fig. 4A). While P*_algZ-_lacZ* reporter activity in the VBS1 mutant was identical to wild-type, mutations in VBS2 and VBS3 significantly lowered activity (*P < *0.01 and *P < *0.0001, respectively) (Fig. 4B). In contrast to the reporter assays, mutation of the VBS1 site resulted in loss of the second shift product, while probes carrying mutations in VBS2 or VBS3 retained the capacity to generate two Vfr-dependent shift products (Fig. 4C). Combinatorial VBS1-2 and VBS2-3 mutations produced small yet additive losses in P*_algZ-_lacZ* reporter activity compared to the VBS2 and VBS3 mutations, respectively (Fig. 4B). Reporter activity of the VBS13 mutant was indistinguishable from that of the single VBS3 mutant. All three double-mutant probes retained the capacity to generate a single Vfr-dependent shift product (Fig. 4C). While P*_algZ_-lacZ* activity was identical in VBS2-3 and VBS1-2-3 mutants (Fig. 4B), all three mutations were required to eliminate Vfr binding by EMSA (Fig. 4C). Activity of the wild-type P*_algZ_*-*lacZ* reporter in a *vfr* mutant was indistinguishable from that of a wild-type strain carrying the VBS1-2-3 mutant P*_algZ_*-*lacZ* reporter (Fig. 4D). Furthermore, deletion of *vfr* in the VBS1-2-3 mutant P*_algZ_*-*lacZ* reporter strain caused no further loss in reporter activity and activity could not be restored by plasmid based *vfr* expression (pPa-*vfr*) (Fig. 4D).

Based on the shift patterns, it appears that Vfr binding to VBS2 and that to VBS3 are mutually exclusive (i.e., binding to one excludes binding to the other), with VBS2 being preferentially occupied when both intact sites are available (Fig. 4C, lanes 2 and 3 versus 4 and 5). Binding to VBS2 or VBS3 was noncompetitive with VBS1. Surprisingly, mutation of VBS3 results in a shift pattern most similar to that of the wild-type probe, but mutation of VBS3 showed the greatest reduction of P*_algZ_* reporter activity *in vivo* (Fig. 4B and C). In general, the EMSA results suggest that all three binding sites are functional; however, the *in vitro* binding patterns are not consistent with promoter activity, indicating that additional factors may contribute to promoter activity *in vivo*. Taken together, these results indicate that all three candidate sites support cAMP-dependent Vfr binding within the *algZ* promoter region and that VBS3 is the primary site involved in Vfr regulation of P*_algZ_* promoter activity. While VBS1 appears to have little or no influence on *algZ* promoter activity, full activity requires both VBS2 and VBS3.

### Both Vfr and AlgZR are required for expression of the *fimU* operon.

**FIG 5:**
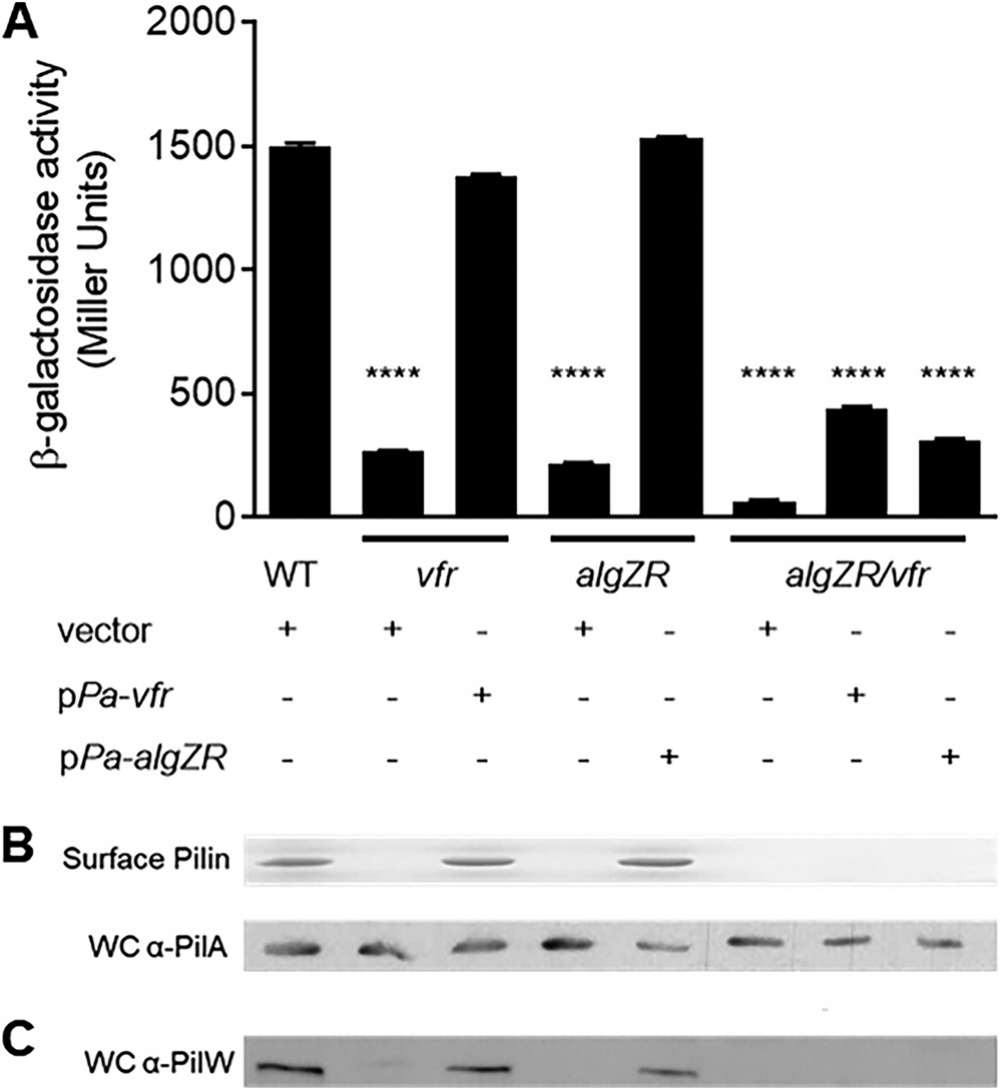
Both AlgR and cAMP/Vfr are required for *fimU* transcription and Tfp production. (A) The wild-type (WT) or indicated mutant strains containing either vector, pPa-*vfr*, or pPa-*algZR* expression plasmids were grown as previously described. Values are means and SEM (*n *≥ 3). The value for the *algZR/vfr* mutant containing any expression plasmid was statistically different from that of the WT strain (****, *P* ≤ 0.0001) as determined by one-way ANOVA with multiple comparisons using Bonferroni’s correction. (B) Coomassie blue-stained SDS-PAGE gel showing pilin from purified pilus fractions isolated from the WT and indicated mutant strains (top) and immunoblot of whole-cell lysates probed with α-PilA antibody (bottom). (C) Immunoblot of whole-cell lysates probed with PilW-specific antiserum. An uncropped blot showing equal protein loading is shown in Fig. S1B.

Based on the findings above, we hypothesized that cAMP and Vfr may indirectly regulate expression of the *fimU* operon by controlling *algZ* expression. This was tested by conducting a series of epistasis experiments. Plasmid-based expression of *algZR* (pPa-*algZR*) was sufficient to restore P*_fimU-_lacZ* reporter activity to wild-type levels in an *algZR* mutant background but not in an isogenic *vfr algZR* triple mutant (Fig. 5A). Similarly, plasmid-expressed *vfr* restored P*_fimU-_lacZ* activity in a *vfr* mutant but not in the *vfr algZR* mutant (Fig. 5A). Consistent with these results, plasmid-based expression of either *vfr* or *algZR* in the *vfr algZR* mutant failed to restore PilW production (Fig. 5C) or Tfp production, as measured by the recovery of pilin in sheared surface fractions (Fig. 5B, top), despite similar levels of pilin in whole-cell lysates (Fig. 5B, bottom). These results indicate that the cAMP/Vfr and AlgZR systems do not act in a strictly linear pathway to control *fimU* operon expression. Overall, these results suggest that cAMP/Vfr and AlgZR are both required for maximal control of *fimU* promoter activity.

### cAMP/Vfr directly regulates transcription of the *fimU* operon.

**FIG 6:**
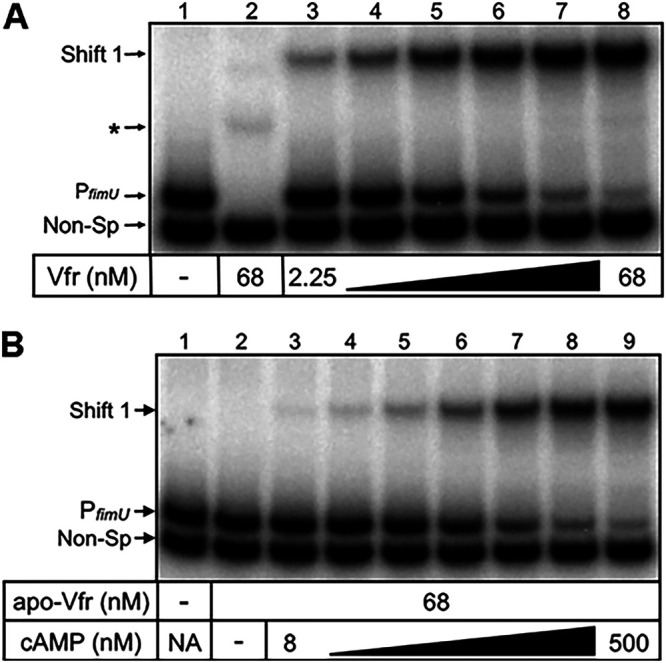
cAMP is required for optimal Vfr binding to P*_fimU_.* (A) Specific (P*_fimU_*, lanes 1 and 3 to 8) and nonspecific (Non-Sp, lane 2) probes (0.25 nM) were incubated in the absence (lane 1) or presence (lanes 2 to 8) of various concentrations of cAMP-Vfr (2.25 to 68 nM) for 15 min followed by electrophoresis and phosphorimaging. The nonspecific probe shift is indicated by an asterisk. (B) Apo-Vfr (68 nM) incubated with specific (P*_fimU_*) and nonspecific (Non-Sp) probes (0.25 nM) in the absence (lane 2) or presence (lanes 3 to 9) of increasing concentrations of cAMP for 15 min followed by electrophoresis and phosphorimaging.

As expression of the *fimU* operon was dependent on both AlgZR and Vfr, we hypothesized that Vfr may also have a direct role in regulating transcription of the *fimU* operon. To test this hypothesis, we assessed Vfr binding to the *fimU* promoter region (Fig. 6A) by EMSA. Shifting of the P*_fimU_* probe was observed with increasing concentrations of cAMP-saturated Vfr (Fig. 6A, lanes 3 to 8). In contrast, apo-Vfr did not shift the P*_fimU_* probe (Fig. 6B, lane 2), but shifting was restored by the addition of cAMP to the binding reaction mixture (Fig. 6B, lanes 3 to 9), thus demonstrating that Vfr requires cAMP for binding to the P*_fimU_* probe.

To identify the Vfr binding site within the *fimU* promoter region, we performed DNase I footprinting. cAMP-saturated Vfr altered the DNase I cleavage pattern of a 41-bp sequence within the P*_fimU_* promoter probe (Fig. S4), corresponding to nucleotides −142 to −102 relative to the *fimU* translational start codon. Enhanced cleavage sites were detected at nucleotides −127 and −117 (Fig. S4). The spacing of DNase I hypersensitivity sites within P*_fimU_* is consistent with the previously reported Vfr footprints of the P*_fleQ_*, P*_lasR_*, P*_toxR_*, P*_regA_*, P*_cpdA_*, and P*_vfr_* promoters (44, 52–56).

**FIG 7:**
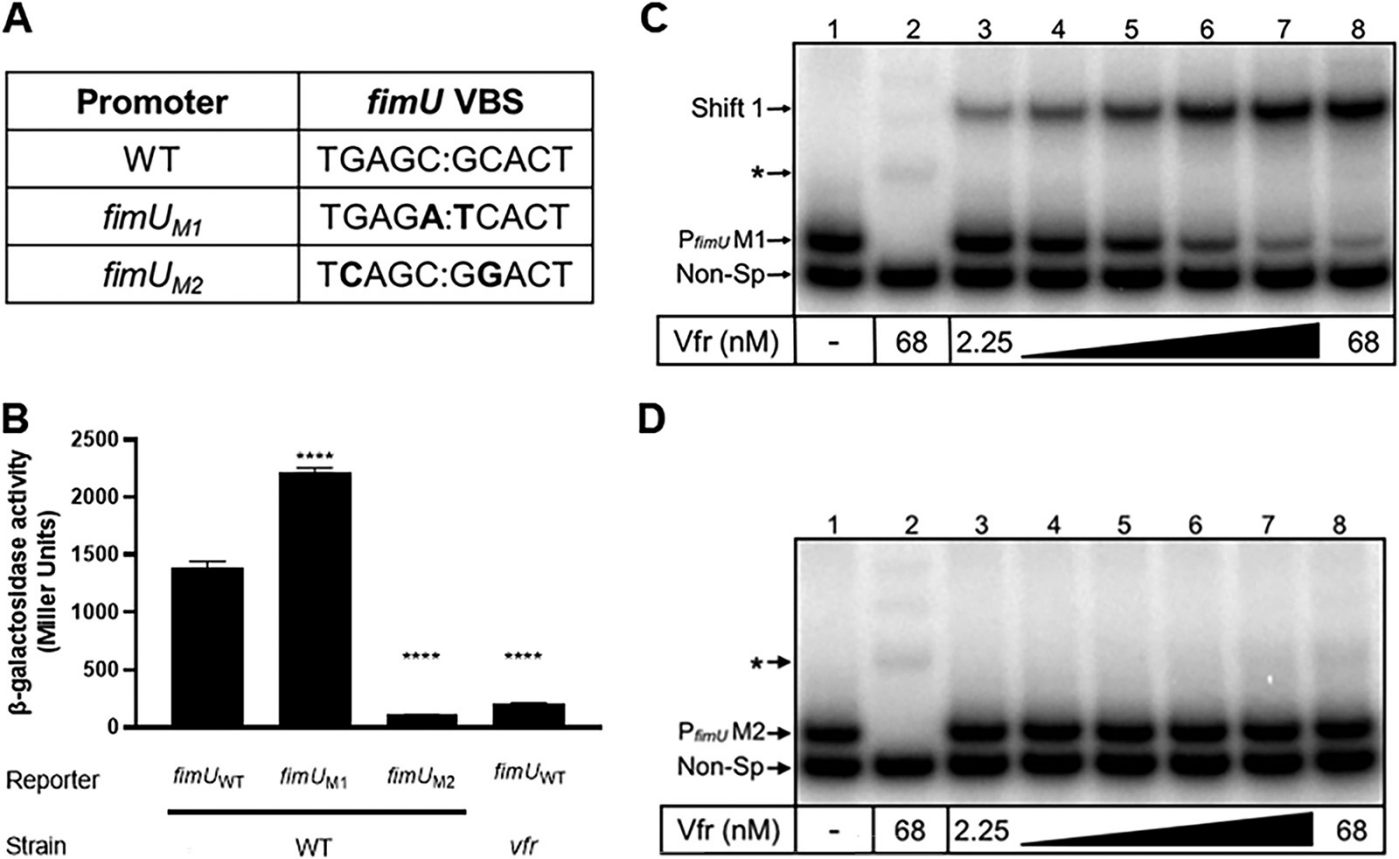
Mutation of the putative Vfr binding site influences *fimU* transcription. (A) Site-directed mutagenesis of the putative Vfr binding half-sites within the *fimU* promoter region. The base changes used to create two mutated versions of the *fimU* promoter (*fimU*_M1_ and *fimU*_M2_) are indicated in bold type. P*_fimU_*_M1_ contains mutations to make the sequence more closely resemble the VCS reported by Fuchs et al. (44). P*_fimU_*_M2_ contains mutations predicted to disrupt Vfr binding. (B) Promoter activity in strains containing either the wild-type *fimU* promoter reporter (P*_fimU_*) or one of the two mutated *fimU* promoter reporters (P*_fimU_*_M1_ or P*_fimU_*_M2_), as measured by β-galactosidase assay. Reporter constructs were in either a wild-type (WT) or *vfr* mutant strain background. Values are means and SEM) (*n *≥ 3). Values for all of the mutants were significantly different (*P* ≤ 0.0001) when compared pairwise to that of the wild-type strain using one-way ANOVA with multiple comparisons using Bonferroni’s correction. (C and D) EMSA analysis of P*_fimU_* probes containing mutations indicated in Fig. 7A. The probes P*_fimU_*M1 (C) and P*_fimU_*M2 (D) were incubated in the absence (lane 1) or presence (lanes 2 to 8) of various concentrations of cAMP-Vfr (2.25 to 68 nM) for 15 min followed by electrophoresis and phosphorimaging. The nonspecific probe shift is indicated by an asterisk.

To confirm the Vfr binding site within the *fimU* promoter, we performed site-directed mutagenesis to alter the predicted half-sites (Fig. 7A). Initially, we mutated the half-sites in the P*_fimU_* transcriptional reporter to more closely mirror the Vfr consensus half-sites (TGAGC→TGAG**A**) and (GCACT→**T**CACT). The resulting reporter (P*_fimU_*_M1_-*lacZ*) resulted in a slight but significant increase in reporter activity relative to the wild-type P*_fimU_*-*lacZ* reporter (Fig. 7B). Mutating the P*_fimU_* Vfr half-sites such that they were more divergent from the consensus half-sites (TGAGC→T**C**AGC and GCACT→G**G**ACT) in the P*_fimU_*_M2_-*lacZ* reporter (Fig. 7A) resulted in a significant decrease in reporter activity compared to P*_fimU_*-*lacZ* (Fig. 7B). The P*_fimU_*_M2_ mutation had the same effect on promoter activity as a *vfr* mutant (Fig. 7B).

To determine whether mutations in the predicted Vfr binding site of P*_fimU_* altered DNA recognition by Vfr, we generated DNA probes corresponding to P*_fimU_*_M1_ and P*_fimU_*_M2_ (Fig. 7A) and assessed Vfr binding by EMSA. As expected, cAMP-saturated Vfr shifted the P*_fimU_*_M1_ probe with no apparent change in binding affinity compared to the wild-type probe (Fig. 6A and 7C). In contrast, cAMP-Vfr failed to bind P*_fimU_*_M2_ (Fig. 7D). Taken together, these results identify the Vfr binding site within the *fimU* promoter and support the finding that cAMP-bound Vfr binds directly to the *fimU* promoter region to activate transcription.

### Vfr and AlgR simultaneously bind the *fimU* promoter.

**FIG 8:**
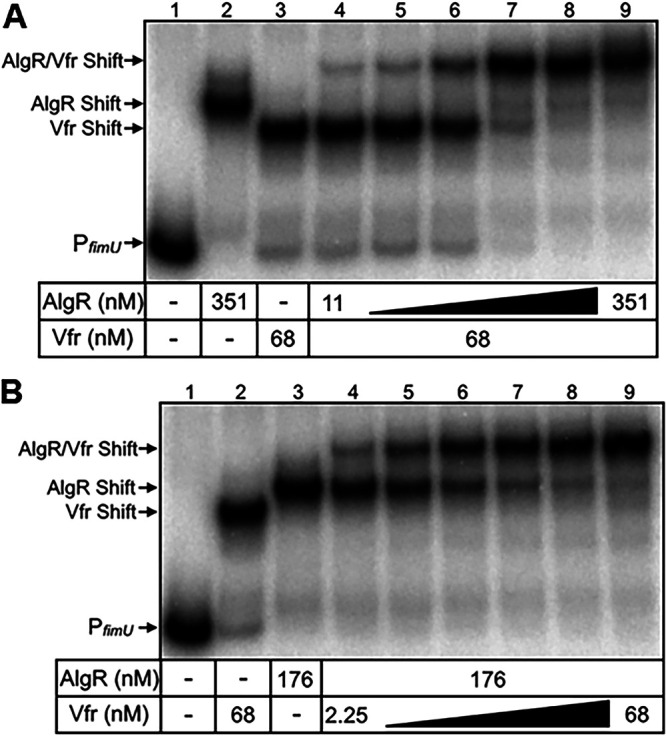
AlgR and Vfr bind to the P*_fimU_*_WT_ probe. (A) P*_fimU_*_WT_ probe incubated in the absence (lane 1) or presence (lanes 2 to 9) of AlgR and Vfr. Lanes 2 and 3 show the concentrations of AlgR and Vfr, respectively resulting in ∼90% shifting of the P*_fimU_*_WT_ probe. Lanes 4 to 9 depict incubation of the P*_fimU_*_WT_ probe and Vfr (68 nM) with increasing concentrations of AlgR (11 to 351 nM). Reactions were conducted in the presence of 50 mM PAM. (B) P*_fimU_*_WT_ probe incubated as for panel A, except that the AlgR concentration was held constant (176 nM) while cAMP-saturated Vfr was added at increasing concentrations (2.25 to 68 nM). Shifted complexes consisting of *fimU* with each of the individual proteins (Vfr shift and AlgR shift) or both proteins (AlgR/Vfr shift) are indicated. Reactions were conducted in the presence of 50 mM PAM.

AlgR and Vfr are both necessary for activation of the *fimU* promoter (Fig. 5) and capable of independently binding to the *fimU* promoter region (Fig. 2 and 6). To determine whether AlgR and Vfr simultaneously bind the *fimU* promoter and whether binding is cooperative, we assessed the binding kinetics by EMSA. The P*_fimU_* probe was incubated with a saturating concentration of cAMP-saturated Vfr and increasing concentrations of AlgR in the presence of PAM (Fig. 8A). Addition of cAMP-saturated Vfr resulted in the expected P*_fimU_* shift product (Fig. 8A, lane 3). The further addition of AlgR resulted in the formation of a second discrete concentration-dependent supershift product (Fig. 8A, lanes 4 to 9), indicating that both proteins simultaneously bound the P*_fimU_* probe. The concentration of AlgR required to shift the P*_fimU_* probe in the presence of Vfr (supershift; Fig. 8A) was similar to the concentration required to shift the probe in the absence of Vfr (Fig. 2C), indicating that Vfr did not significantly influence AlgR binding.

To determine the impact of AlgR binding on Vfr recruitment, the P*_fimU_* probe was incubated with a saturating amount of AlgR (in the presence of PAM) and increasing concentrations of cAMP-saturated Vfr (Fig. 8B). AlgR formed the expected shift product (Fig. 8B, lane 3), and addition of Vfr resulted in formation of a second discrete shift product. Overall, these results demonstrate that both AlgR and Vfr can simultaneously bind the *fimU* promoter, and binding of either protein appears to be largely independent of the other. However, simultaneous recognition of the *fimU* promoter by AlgR and Vfr has a positive and combined effect on *fimU* transcription (Fig. 5A).

### *fimU* operon complementation does not restore the Tfp defect of *vfr* mutants.

**FIG 9:**
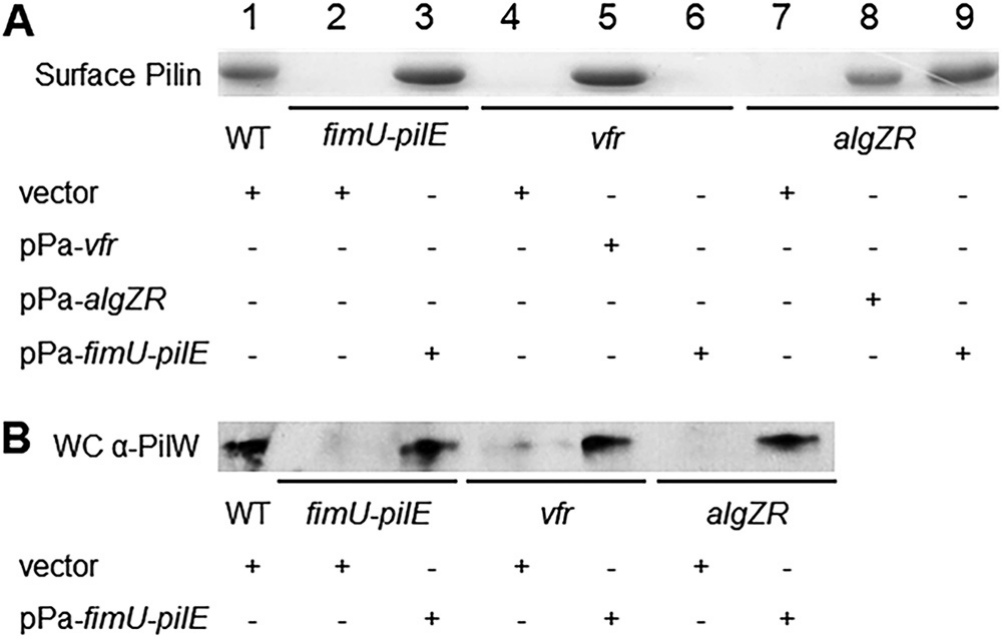
The *fimU* operon is not sufficient to restore the Tfp defect of a *vfr* mutant. (A) Tfp production as determined by Coomassie blue-stained SDS-PAGE gels of purified pilus fractions from WT and indicated mutant strains. (B) Immunoblots from whole-cell lysates of WT and indicated mutant strains probed with PilW-specific antiserum. An uncropped blot showing equal protein loading is shown in Fig. S1C.

To determine whether the Tfp defect of *algZR* and *vfr* mutants is attributed solely to the lack of *fimU* transcription, we complemented the *algZR* and *vfr* mutants with plasmid-based expression of the entire *fimU* operon (pPa-*fimU-pilE*). When expressed under conditions that restored Tfp production in a *fimU-pilE* mutant (Fig. 9A, lane 3), pPa-*fimU-pilE* was sufficient to restore Tfp production in the *algZR* mutant (Fig. 9A, lane 9) but not in the *vfr* mutant (Fig. 9A, lane 6). As a further control, we confirmed that plasmid-based expression of *vfr* (pPa-*vfr*) and *algZR* (pPa-*algZR*) was sufficient to restore Tfp production in the *vfr* and *algZR* mutants, respectively (Fig. 9A, lanes 5 and 8). pPa-*fimU-pilE* complementation also restored PilW expression to both the *vfr* and *algZR* mutants (Fig. 9B). Taken together, these results suggest that the Tfp defect in the *algZR* mutant can be attributed solely to AlgZR regulation of the *fimU* operon, whereas the Tfp defect of *vfr* mutants involves regulation of other factors in addition to the *fimU* operon.

## DISCUSSION

The regulatory complexity of P. aeruginosa Tfp production and function is not surprising given the number of components involved, their various functions, and their genetic arrangement. There are distinct mechanisms governing the structural components of Tfp as well as the factors involved in processing and assembly, all of which may be regulated at the level of transcription and/or function (e.g., fiber extension/retraction and surface sensing). While this study builds upon the work of multiple investigators (33, 35, 52, 57), we present several novel results that contribute to the understanding of Tfp regulation. We identified two independent, but linked, regulatory systems involving the transcription factors AlgR and Vfr that control expression of the *fimU* operon. These findings suggest a mechanistic explanation for the Tfp biogenesis and twitching motility defects of *algZR* and *vfr* mutants. However, as shown in Fig. 9, the Tfp defect in the *vfr* mutant is not solely attributed to *fimU* operon control, as complementation of *fimU* does not restore Tfp expression. The possibility that Vfr controls additional factors necessary for Tfp biogenesis and expression is also supported by microarray analysis, which identified several Tfp biogenesis-related genes with altered expression in a *vfr* mutant (42). Several categories of Tfp biogenesis-related genes displayed altered expression levels in the microarray study, including (i) Chp chemosensory system genes (*pilG*, *pilJ*, and *pilK*), which regulates twitching motility and cAMP production; (ii) essential pilus biogenesis machinery components (*pilNOPQ*, *pilZ*, and *pilU*); (iii) additional regulators of Tfp biogenesis and/or function (*fimV* and *pilS*); and (iv) *pilD*, the prepilin peptidase. Altered expression of most of these genes (except *pilS*) does not affect intracellular pilin levels but instead controls Tfp production at the level of assembly and/or function and could contribute to the Tfp biogenesis and twitching motility defect of *vfr* mutants.

A previous bioinformatic analysis predicted a Vfr binding site in the *algZ* promoter and showed that Vfr binds to a P*_algZ_* probe by EMSA, suggesting that Vfr regulates *fimU* indirectly through the AlgZR system (52). We demonstrated that cAMP/Vfr positively regulates both the *fimU* and *algZ* promoters, revealing both direct and indirect modes of *fimU* transcriptional control. Although Kanack et al. (52) observed a single Vfr-P*_algZ_* shift product via EMSA, we visually identified two additional Vfr binding sites within the *algZ* promoter and observed two shift products in our *in vitro* analysis. We showed that mutation of all three sites is required for maximal loss of P*_algZ_*-*lacZ* activity and Vfr binding to a P*_algZ_* via EMSA. Although individual mutations in VBS2 and VBS3 but not VBS1 resulted in reduced P*_algZ_-lacZ* activity, combinatorial VBS1-2 and VBS2-3 mutations exhibited additive reporter activity decreases. Our results are consistent with the microarray analysis of a *vfr* mutant, which initially indicated Vfr-dependent regulation of *algZ* expression (42).

Despite the fact that the *fimU* promoter lacked a predicted Vfr binding site, our study provides the following evidence for Vfr-dependent regulation of *fimU* transcription: (i) P*_fimU_*-*lacZ* reporter activity was significantly decreased in both *cyaAB* and *vfr* mutants, (ii) Vfr shifted a P*_fimU_* probe in a concentration- and cAMP-dependent manner *in vitro*, and (iii) mutation to the Vfr half-sites identified by DNase I footprinting diminished P*_fimU_*-*lacZ* reporter activity and abolished binding of Vfr *in vitro*. In fact, the VBS (5′-AACG**TGAGC**TAT:GCA**GCACT**T) we identified within the *fimU* promoter region shares reasonable consensus with the previously identified VCS (5′-ANWW**TGNGA**WNY:AGW**TCACA**T). The upstream Vfr half-site (TGAGC) shares considerable conservation with the upstream VCS half-site (TGNGA), while the downstream *fimU* Vfr half-site (GCACT) deviates significantly from the downstream VCS half-site (TCACA). Given the results of previous studies that have characterized the Vfr binding sites for several Vfr-dependent targets (44, 52), it is clear that Vfr binding sites display significant variability, and thus, it is not surprising that several Vfr-dependent genes are directly regulated by Vfr activity that were not previously identified via bioinformatics.

Activation of alginate production by AlgR is AlgZ independent and therefore likely phosphorylation independent (36). However, since both AlgZ and AlgR control Tfp function, albeit by different assays in different strains, it suggests that AlgR phosphorylation by AlgZ is required for Tfp formation (34, 35, 58). AlgR phosphorylation impacts regulation of the iron-scavenging molecules pyocyanin and pyoverdine, further highlighting the role of different AlgR phosphorylation states (59).

Previous work demonstrated that AlgR positively regulates the *fimU* operon and identified two AlgR binding sites within the *fimU* promoter region (35). We extended these findings by analyzing the Tfp phenotype and P*_fimU_*-*lacZ* reporter activity of mutants in which either *algZ* or *algR* was inactivated. Both *algZ* and *algR* single mutants were defective for Tfp production and displayed P*_fimU_*-*lacZ* reporter activity equivalent to an *algZR* double mutant, suggesting that AlgZ activity is required for AlgR regulation of *fimU.* Although previous studies found that AlgR can be phosphorylated *in vitro* using the Escherichia coli CheA kinase as a phosphodonor (34, 60), these studies did not directly address the role of AlgR phosphorylation in activation of the *fimU* operon. Our *fimU* promoter EMSA studies using PAM as a phosphodonor resulted in a significant increase in AlgR binding affinity. Taken together, these results provide strong evidence that AlgR phosphorylation is necessary for regulation of the *fimU* operon. Due to the absence of conserved kinase motifs (D/F and G boxes) required for ATP binding, it has been suggested that AlgZ does not function as a sensor kinase but may instead function as a phosphatase (36). However, experimental results supporting either function have been limited to the characterization of *algZ* mutant phenotypes.

Based on response regulators homologous to AlgR, we predicted that phosphorylation would affect DNA binding (61). In our study, unphosphorylated AlgR bound the P*_fimU_* probe at high concentrations, and phosphorylation of AlgR dramatically reduced the amount of AlgR required to shift the P*_fimU_* probe. This result suggests that, regardless of phosphorylation status, AlgR recognizes the *fimU* promoter, but phosphorylation serves to enhance DNA affinity. Interestingly, promoters known to be regulated by unphosphorylated AlgR are associated with chronic infection phenotypes (*algC* and *algD*) (62), whereas phosphorylated AlgR regulates genes more often associated with acute phenotypes (*fimU* operon and *hcnA*) (35, 63). Thus, AlgR phosphorylation may serve as a signal responsible for the commitment to a particular virulence phenotype.

Given the requirement of both transcriptional regulators Vfr and AlgR for activation of the *fimU* promoter, we attempted to determine whether the binding of either required the presence of the other. We determined that phosphorylated AlgR and cAMP-saturated Vfr are capable of binding the P*_fimU_* probe simultaneously. While binding of either Vfr or AlgR to the *fimU* promoter was largely independent of the presence of the other, there is a combined effect of AlgR and Vfr on activation of the *fimU* promoter. Collectively, the results of our study demonstrate that AlgR activates the *fimU* operon in a phosphorylation-dependent manner, presumably via phosphorylation by the cognate putative sensor kinase AlgZ. Furthermore, we extend previous phenotypic observations implicating Vfr in Tfp expression by demonstrating that Vfr directly regulates the *fimU* operon in a cAMP-dependent manner.

## MATERIALS AND METHODS

### Bacterial strains, growth conditions, and plasmids.

The bacterial strains and plasmids used in this study are described in Table S1. Bacteria (E. coli and P. aeruginosa) were routinely grown at 37°C in LB medium. pMMB-based expression plasmids were maintained in P. aeruginosa with 150 μg/mL carbenicillin (Cb), except where indicated. Bacterial growth in liquid culture was assessed by optical density at 600 nm (OD_600_). Assays for β-galactosidase activity were performed as previously described (56).

10.1128/mbio.03696-21.5 TABLE S1 Strains and plasmids used in this study. Download Table S1, DOCX file, 0.05 MB. Copyright © 2022 Coggan et al. 2022 Coggan et al. https://creativecommons.org/licenses/by/4.0/ This content is distributed under the terms of the Creative Commons Attribution 4.0 International license.

With the exception of *algZ*, all gene deletion alleles were constructed, introduced onto the chromosomes of the appropriate P. aeruginosa strains, and confirmed as previously described (42). The *algZ* gene was disrupted by converting the translational start codon into a stop codon (ATG→TGA), using splice PCR, to avoid ablating a previously identified *algR* promoter within the 3′ region of the *algZ* coding sequence (51). All other point mutations were generated by site-directed mutagenesis using a commercially available kit (QuikChange II; Agilent Technologies). See Table S2 for primer sequences.

10.1128/mbio.03696-21.6 TABLE S2 Primers used in this study. Mutated sites are indicated by italics. EcoRI and BamHI restriction sites are underlined, and *att*B1 and *att*B2 tails are in boldface. Download Table S2, DOCX file, 0.05 MB. Copyright © 2022 Coggan et al. 2022 Coggan et al. https://creativecommons.org/licenses/by/4.0/ This content is distributed under the terms of the Creative Commons Attribution 4.0 International license.

P*_algZ_* and P*_fimU_* transcriptional reporters were constructed by PCR amplifying the *fimU* promoter region (−311 to +153 bp relative to the *fimU* transcriptional start site) and the *algZ* promoter region (−283 to +107 bp relative to the *algZ* transcriptional start site) from *P. aeruginosa* strain K (PAK) chromosomal DNA with EcoRI and BamHI restriction sites and *att*B1 and *att*B2 tails for cloning into pDONR201 via Gateway cloning (see Table S2 for primers). Promoter fragments were digested from pDONR201 using EcoRI and BamHI, ligated into mini-CTX-*lacZ* and introduced into P. aeruginosa as previously described (44, 64).

### Phage susceptibility assay.

Susceptibility to the pilin-specific phage PO4 was assayed using the double-agar method as previously described (50). Briefly, overnight cultures of each strain were mixed with molten LB with 0.75% agar (top agar) and poured over standard LB agar plates. Phage were serially diluted from an initial phage stock of 10^8^ PFU/mL. After the top agar solidified, 10 μL of each phage dilution, as well as a broth control, was spotted onto the top agar. Plates were incubated at 37°C overnight and imaged. A mutant of *pilA* and its complement were included as controls. IPTG (isopropyl-β-d-thiogalactopyranoside; 250 μM) was added for the *pilA* complement (pPa-*pilA*).

### Electron and immunofluorescence microscopy.

Transmission electron microscopy (TEM) was performed as described previously (65), with the exception that grids were placed on a drop of bacterial suspension at 22°C for 10 min and the samples were stained with an aqueous 0.5% ammonium molybdate solution for 10 min before being viewed in a Philips CM100 transmission electron microscope. Immunofluorescence (IF) microscopy was performed as described previously (48), except that the P. aeruginosa strains were grown to an OD_600_ of 0.2 prior to incubating the bacteria on poly-l-lysine-coated glass coverslips. P. aeruginosa pilin-specific antiserum (gift from E. C. Gotschlich, Rockefeller University) was used as a primary antibody for Tfp labeling, followed by an Alexa Red 594-conjugated goat anti-rabbit IgG (Molecular Probes). P. aeruginosa cells were stained with 4′-6-diamidino-2-phenylindole (DAPI) at 1 μg/mL in Mowiol mounting medium (Sigma) containing 2% 1,4-diazabicyclo(2)octane (DABCO) prior to viewing with a Nikon Eclipse C400 fluorescence microscope.

### Isolation of surface Tfp.

Surface pilin was isolated as previously described (8). Briefly, P. aeruginosa was grown on LB agar plates until confluent. Bacteria were collected and suspended in 1 mL 0.15 M NaCl, 0.2% formaldehyde and vortexed vigorously for 1 min to release surface Tfp. Bacterial cells were removed by centrifugation at 12,000 × *g* for 5 min, and the total protein content of the bacterial pellet was determined by a Bradford assay. Supernatants were transferred to new tubes, adjusted to 0.1 M MgCl_2_, and incubated at 4°C for 12 h. Following centrifugation at 12,000 × *g* for 5 min, the resulting Tfp pellets were suspended in sodium dodecyl sulfate-polyacrylamide gel electrophoresis (SDS-PAGE) sample buffer, normalized based on total protein in the bacterial pellet fraction, and separated by SDS-PAGE (18% polyacrylamide), and pilin was visualized by Coomassie blue staining.

### Immunoblotting.

Whole-cell lysates were prepared from bacteria grown in LB broth to mid-exponential growth phase (OD_600_ = 1). Strains harboring plasmids were grown in the presence of 30 μg/mL Cb and the indicated amount of IPTG. Bacteria were collected by centrifugation, suspended in 100 μl of SDS-PAGE sample buffer, incubated at 95°C for 10 min, and normalized based on total protein. The lysate was diluted ∼1:10, separated on SDS-polyacrylamide gels (18% for pilin, 7.5% for PilW), and transferred to nitrocellulose or polyvinylidene difluoride (PVDF) membranes, respectively. Membranes were probed with PKL1 anti-pilin mouse monoclonal antibody (66) (1:30,000 dilution; gift from Randall Irvin, University of Alberta) or anti-PilW rabbit serum (23) (1:8,000 dilution). Horseradish peroxidase-conjugated secondary antibodies were used for the detection of specific antibody-antigen complexes. Blots were developed with chemiluminescence reagents (Millipore) and visualized via autoradiography.

### EMSA and DNase I footprinting.

DNA promoter probes were generated by PCR using the indicated oligonucleotides and end labeled using 10 μCi of [γ-^32^]ATP (GE Healthcare) and 10 U of T4 polynucleotide kinase (New England Biolabs). EMSAs were performed as previously described (67). Briefly, probes (0.25 nM each) were incubated in binding buffer (10 mM Tris [pH 7.5], 50 mM KCl, 1 mM EDTA, 1 mM dithiothreitol [DTT], 5% glycerol, and 100 μg/mL bovine serum albumin) containing 5 μg/mL poly(dI-dC) for 5 min at 25°C. cAMP-saturated Vfr, apo-Vfr, or His_6_-AlgR protein were added as indicated in the figure legends to a final volume of 20 μL and incubated for an additional 15 min at 25°C. Where indicated (in the figure legends), 50 mM PAM and 1 mM MgCl_2_ was incubated with His_6_-AlgR. Samples were subjected to electrophoresis on a 5% polyacrylamide glycine gel (10 mM Tris [pH 7.5], 380 mM glycine, 1 mM EDTA) at 4°C. Imaging and data analyses were performed using an FLA-700 Phosphorimager (Fujifilm) and MultiGauge v3.0 software (Fujifilm). EMSAs were repeated a minimum of two times, and representative gels are shown.

Single end-labeled probe for DNase I footprinting was generated by PCR and labeled with ^32^P as previously described (56). Labeled probe (10 fmol) was incubated with 5 μg/mL poly(dI-dC) (Sigma) in DNase I reaction buffer (10 mM Tris [pH 8.0], 50 mM KCl, 2 mM MgCl_2_, 0.5 mM DTT, 100 μg/mL bovine serum albumin, 10% glycerol with purified Vfr as indicated in a final reaction volume of 25 μl. Reaction mixtures were incubated for 15 min at 25°C, fractionated on denaturing sequencing gel, and analyzed using a FLA-700 Phosphorimager.

### Protein purification.

Apo-Vfr and cAMP saturated Vfr were generated as previously described (42, 54). The hexa-histidine tagged AlgR expression plasmid was constructed as follows. The *algR* coding sequence was amplified from PAK genomic DNA using primers. The resulting PCR fragment was gel purified (Qiagen), digested with NdeI and BamHI, and ligated into the corresponding sites in pET28a. After confirmation via sequencing, the resulting plasmid was transformed into E. coli BL21(DE3) strains for protein overexpression.

An overnight culture of E. coli strain BL21 carrying the pET28a-AlgR overexpression construct was subcultured (1:100) into 1 L LB containing 25 μg/mL kanamycin and grown at 37°C to an OD_600_ of ∼0.8. The flask was then chilled to room temperature (RT), induced with 0.5 mM IPTG, and cultured at 23°C overnight on a rotary shaker (∼30 rpm). Cells were collected and washed twice via resuspension in 40 mL of binding buffer (50 mM NaH_2_PO_4_ [pH 8.0], 300 mM NaCl, 20 mM imidazole). Cells were harvested, washed twice with binding buffer, and suspended in a 20-mL binding buffer. Following a 30-min incubation with 0.5 mg/mL lysozyme on ice, the cells were lysed by sonication and centrifuged at 10,000 × *g* for 30 min to remove cellular debris. The soluble fraction was then incubated with 3 mL of Ni^2+^-charged resin at 4°C for 2 h with gentle rotation. The resin was loaded onto a 20-mL Bio-Rad disposable column, washed twice with wash buffer (50 mM NaH_2_PO_4_ [pH 8.0], 300 mM NaCl, 40 mM imidazole), and then eluted with elution buffer (50 mM NaH_2_PO_4_ [pH 8.0], 300 mM NaCl, 100 mM imidazole). Fractions containing significant amounts of 6His-AlgR, as determined by SDS-PAGE analysis, were concentrated using a Centriprep concentrator and subsequently loaded on a Sephadex 75 size elution column. Fractions from a peak corresponding to AlgR (27 kDa) were concentrated to ∼3 mL, loaded into a Slide-a-Lyzer (Pierce), dialyzed twice against 2 L of buffer (20 mM Tris [pH 8.0], 500 mM NaCl, 1 mM DTT, 0.5% Tween 20), and then dialyzed into storage buffer (20 mM Tris [pH 8.0], 500 mM NaCl, 1 mM DTT, 0.5% Tween 20, 50% glycerol). Protein concentration was determined by Bradford assay.
