# Supplementary figures and images for: Global Regulatory Pathways Converge To Control Expression of Pseudomonas aeruginosa Type IV Pili
Source: mBio. 2022 Jan 25;13(1):e03696-21. doi: 10.1128/mbio.03696-21 (PMC8787478; doi:10.1128/mbio.03696-21)

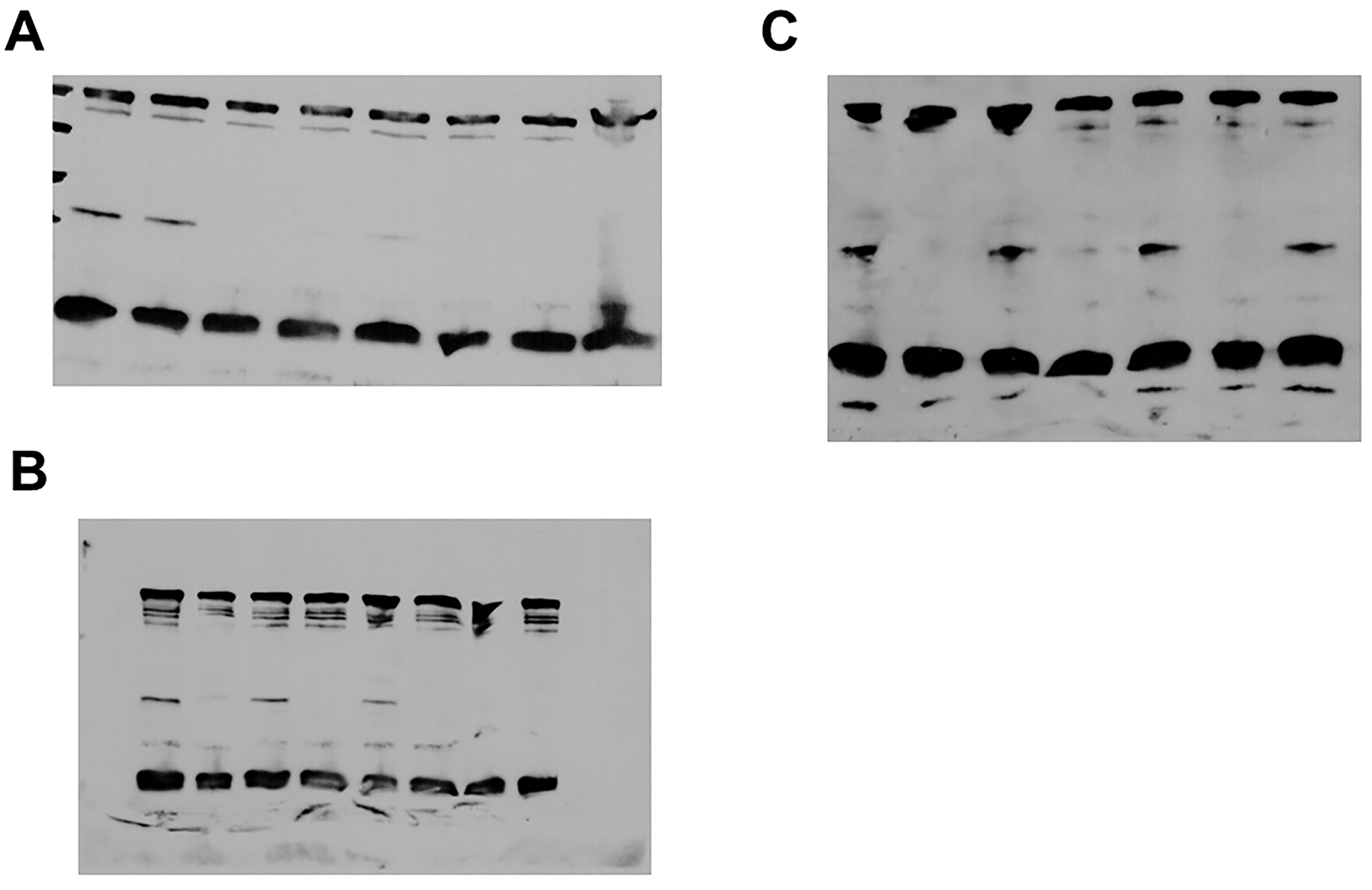

Supplement: FIG S1 [file mbio.03696-21-sf001.tif]

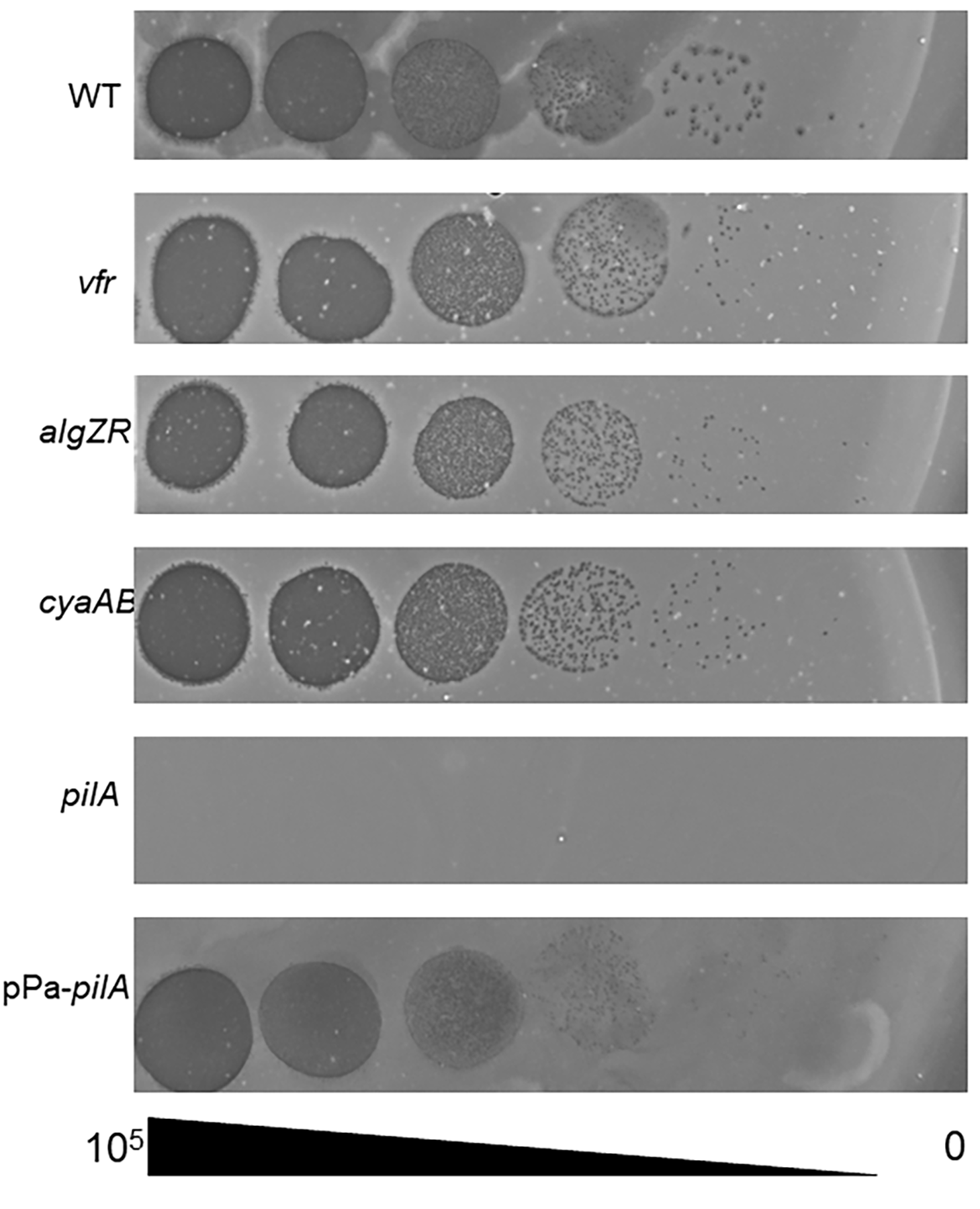

Supplement: FIG S2 [file mbio.03696-21-sf002.tif]

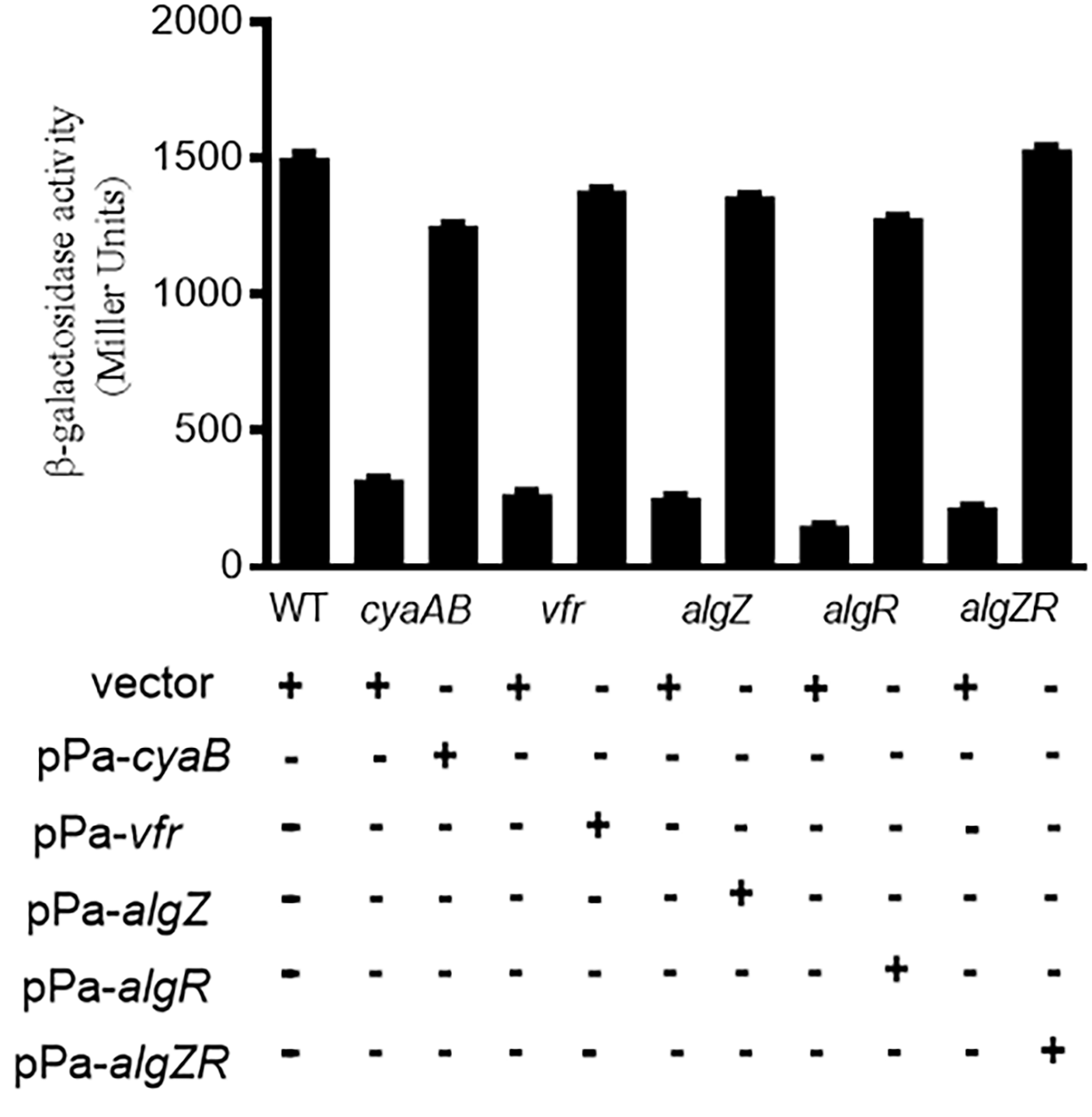

Supplement: FIG S3 [file mbio.03696-21-sf003.tif]

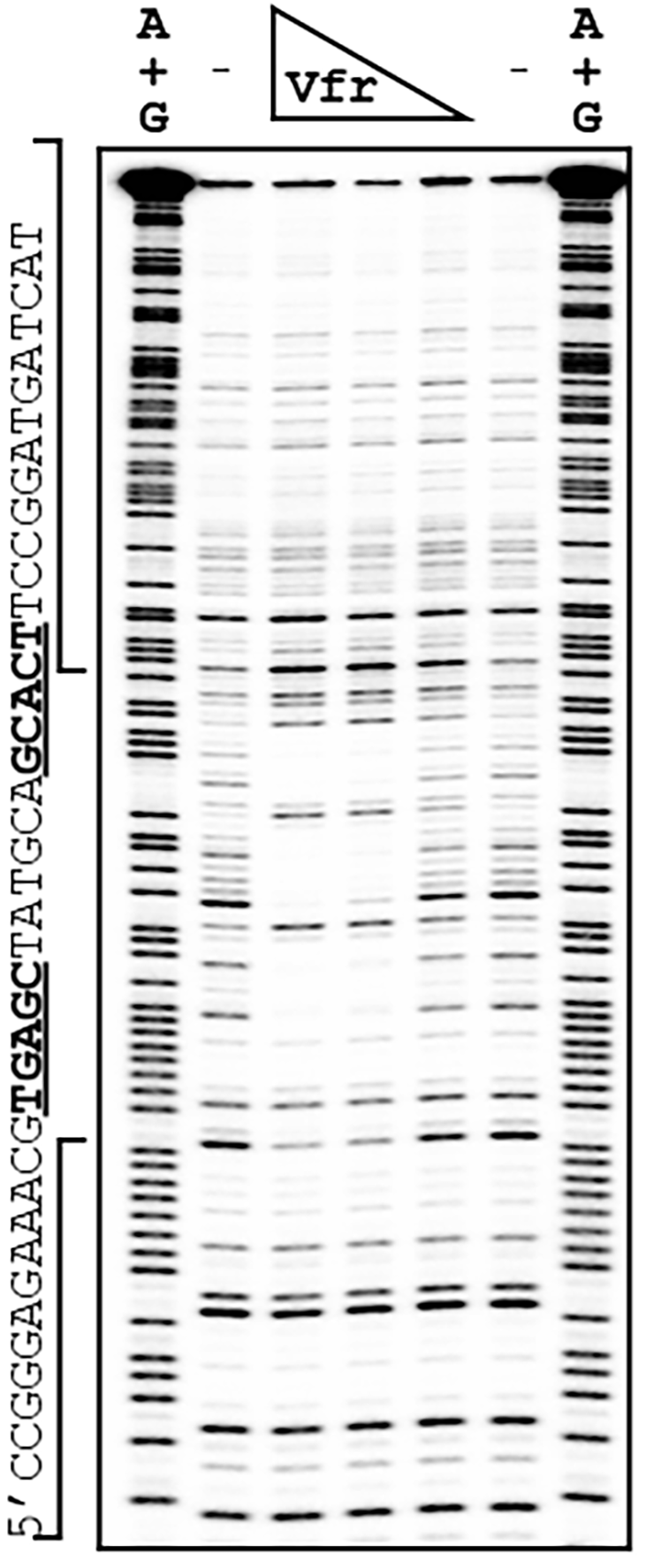

Supplement: FIG S4 [file mbio.03696-21-sf004.tif]
