# Supplementary material for: Global Regulatory Pathways Converge To Control Expression of Pseudomonas aeruginosa Type IV Pili
Source: mBio. 2022 Jan 25;13(1):e03696-21. doi: 10.1128/mbio.03696-21 (PMC8787478; doi:10.1128/mbio.03696-21)
Supplement: TABLE S1 [file mbio.03696-21-st001.docx]

**Supplemental Table 1: Strains and Plasmids used in this study**

| **Strain or plasmid** | **Description or relevant characteristics** | **Reference or source** |
| --- | --- | --- |
| ***P. aeruginosa* strains** |  |  |
| PAK | *P. aeruginosa* strain K, wild type | [(65)](https://sciwheel.com/work/citation?ids=11506173&pre=&suf=&sa=0&dbf=0) |
| PAK::P*fimU-lacZ* | PAK with chromosomal *fimU* promoter reporter | This study |
| PAKΔ*pilA*::P*fimU-lacZ* | *pilA* with chromosomal *fimU* promoter reporter | This study |
| PAKΔ*fimU-pilE*::P*fimU-lacZ* | *fimU-pilVWXY1Y2E* with chromosomal *fimU* promoter reporter | This study |
| PAKΔ*cyaAB*::P*fimU-lacZ* | *fimU-pilVWXY1Y2E* with chromosomal *fimU* promoter reporter | This study |
| PAKΔ*vfr*::P*fimU-lacZ* | *vfr* with chromosomal *fimU* promoter reporter | This study |
| PAKΔ*algZ*::P*fimU-lacZ* | *algZ* with chromosomal *fimU* promoter reporter | This study |
| PAKΔ*algR*::P*fimU-lacZ* | *algR* with chromosomal *fimU* promoter reporter | This study |
| PAKΔ*algZR*::P*fimU-lacZ* | *algZR* with chromosomal *fimU* promoter reporter | This study |
| PAKΔ*algZR*Δ*vfr*::P*fimU-lacZ* | *algZRvfr* with chromosomal *fimU* promoter reporter | This study |
| PAK::P*fimU_M1_-lacZ* | PAK with chromosomal *fimU_M1_* promoter reporter | This study |
| PAK::P*fimU_M2_-lacZ* | PAK with chromosomal *fimU_M2_* promoter reporter | This study |
| PAK::P*algZ-lacZ* | PAK with chromosomal *algZ* promoter reporter | This study |
| PAKΔ*cyaAB*::P*algZ-lacZ* | *cyaAB* with chromosomal *algZ* promoter reporter | This study |
| PAKΔ*vfr*::P*algZ-lacZ* | *vfr* with chromosomal *algZ* promoter reporter | This study |
| PAK::P*algZ_VBS1_-lacZ* | PAK with chromosomal *algZ_VBS1_* promoter reporter | This study |
| PAK::P*algZ_VBS2_-lacZ* | PAK with chromosomal *algZ_VBS2_* promoter reporter | This study |
| PAK::P*algZ_VBS3_-lacZ* | PAK with chromosomal *algZ_VBS3_* promoter reporter | This study |
| PAK::P*algZ_VBS1-2_-lacZ* | PAK with chromosomal *algZ_VBS1-2_* promoter reporter | This study |
| PAK::P*algZ_VBS1-3_-lacZ* | PAK with chromosomal *algZ_VBS1-3_* promoter reporter | This study |
| PAK::P*algZ_VBS2-3_-lacZ* | PAK with chromosomal *algZ_VBS2-3_* promoter reporter | This study |
| PAK::P*algZ_VBS1-2-3_-lacZ* | PAK with chromosomal *algZ_VBS1-2-3_* promoter reporter | This study |
| PAKΔ*vfr*::P*algZ_VBS1-2-3_-lacZ* | *vfr* with chromosomal *algZ_VBS1-2-3_* promoter reporter | This study |
| **Plasmids** |  |  |
| pDONR201 | Gateway cloning vector, Km^r^ | Life Technologies |
| mini-CTX-*lacZ* | Plasmid for chromosomal integration at the ΦCTX  phage site of transcriptional fusions; Tc^r^ | [(66)](https://sciwheel.com/work/citation?ids=2549955&pre=&suf=&sa=0&dbf=0) |
| mini-CTX-P*fimU-lacZ* | *fimU* promoter region (523 bp) in *Eco*RI  and *Bam*HI sites of mini-CTX-*lacZ*; Tc^r^ | This study |
| mini-CTX-P*algZ-lacZ* | *algZ* promoter region (441 bp) in *Eco*RI and *Bam*HI sites of mini-CTX-*lacZ*; Tc^r^ | This study |
| pEX18Gm | Suicide vector, Gm^r^ | [(66)](https://sciwheel.com/work/citation?ids=2549955&pre=&suf=&sa=0&dbf=0) |
| pEXGmΔ*fimU-pile* | pEX18Gm containing *fimU-pilE* deletion allele; Gm^r^ | [(8)](https://sciwheel.com/work/citation?ids=11745833&pre=&suf=&sa=0&dbf=0) |
| pEXGmΔ*pilA* | pEX18Gm containing *pilA* deletion allele; Gm^r^ | [(44)](https://sciwheel.com/work/citation?ids=2263220&pre=&suf=&sa=0&dbf=0) |
| pEXGmΔ*cyaA* | pEX18Gm containing *cyaA* deletion allele; Gm^r^ | [(31)](https://sciwheel.com/work/citation?ids=905434&pre=&suf=&sa=0&dbf=0) |
| pEXGmΔ*cyaB* | pEX18Gm containing *cyaB* deletion allele; Gm^r^ | [(31)](https://sciwheel.com/work/citation?ids=905434&pre=&suf=&sa=0&dbf=0) |
| pEXGmΔ*vfr* | pEX18Gm containing *vfr* deletion allele; Gm^r^ | [(31)](https://sciwheel.com/work/citation?ids=905434&pre=&suf=&sa=0&dbf=0) |
| pEXGmΔ*algZ* | pEX18Gm containing *algZ* deletion allele; Gm^r^ | This study |
| pEXGmΔ*algR* | pEX18Gm containing *algR* deletion allele; Gm^r^ | This study |
| pEXGmΔ*algZR* | pEX18Gm containing *algZR* deletion allele; Gm^r^ | [(39)](https://sciwheel.com/work/citation?ids=11746015&pre=&suf=&sa=0&dbf=0) |
| pMMB67EH | Empty *P. aeruginosa* expression vector; Ap^r^ | [(67)](https://sciwheel.com/work/citation?ids=424320&pre=&suf=&sa=0&dbf=0) |
| pMMBV1GW | Gateway-adapted version of pMMB67EH; Ap^r^ | [(35)](https://sciwheel.com/work/citation?ids=7556515&pre=&suf=&sa=0&dbf=0) |
| pPa-*cyaB* | *P. aeruginosa cyaB* carried on pMMBV2; Ap^r^ | [(35)](https://sciwheel.com/work/citation?ids=7556515&pre=&suf=&sa=0&dbf=0) |
| pPa-*vfr* | *P. aeruginosa vfr* carried on pMMBV1; Ap^r^ | [(44)](https://sciwheel.com/work/citation?ids=2263220&pre=&suf=&sa=0&dbf=0) |
| pPa-*algZ* | *P. aeruginosa algZ* carried on pMMBV1; Ap^r^ | This study |
| pPa-*algR* | *P. aeruginosa algR* carried on pMMBV3; Ap^r^ | This study |
| pPa-*algZR* | *P. aeruginosa algZR* carried on pMMBV1; Ap^r^ | [(41)](https://sciwheel.com/work/citation?ids=7556630&pre=&suf=&sa=0&dbf=0) |
| pPa-*fimU-pile* | *P. aeruginosa fimU-pilE* carried on pMMBV1; Ap^r^ | [(8)](https://sciwheel.com/work/citation?ids=11745833&pre=&suf=&sa=0&dbf=0) |
| Ap^r^, ampicillin resistance marker; Km^r^, kanamycin resistance marker; Gm^r^, gentamicin resistance marker; Tc^r^, tetracycline resistance marker | | |
