# Supplementary material for: Global Regulatory Pathways Converge To Control Expression of Pseudomonas aeruginosa Type IV Pili
Source: mBio. 2022 Jan 25;13(1):e03696-21. doi: 10.1128/mbio.03696-21 (PMC8787478; doi:10.1128/mbio.03696-21)
Supplement: TABLE S2 [file mbio.03696-21-st002.docx]

**Supplemental Table 2: Primers**

| **Primer** | **Sequence 5’-3’** |
| --- | --- |
| **AlgR Protein Purification** |  |
| Nde-AlgR | TACAAAAAAGCAGGCTCATATGAATGTCCTGATTGTCGATGAC |
| BamHI-AlgR | TACAAGAAAGCTGGGTGGATCCTCAGAGCTGATGCATCAGACGCCTGAC |
| **algZ EMSA** |  |
| PalgZ 5’ | CGCGCGGCCTGCGTGACGGGC |
| PalgZ 3’ | GTACGGGCGAAATAAAGCACCGAA |
| **Reporters** |  |
| PfimU 5’ | **TACAAAAAAGCAGGCT**GAATTCTAGAGGGCTGGCTGCTTGCGGAAGAC |
| PfimU 3’ | **TACAAGAAAGCTGGGT**GGATCCTCGCATTGAGTTCCTCGGCGGCGCTCTG |
| PalgZ 5’ | **TACAAAAAAGCAGGCT**GAATTCTCAGCGGTAGAGACGCTTGTCGAAGTC |
| PalgZ 3’ | **TACAAGAAAGCTGGGT**GGATCCTCAGCAGCACCAGGGTGAACAGGGC |
| **fimU Footprinting** |  |
| PfimU5’FPmod | /5AmMC6/GCATACCGTTGCCTGGCAACTGAT |
| PfimU3’ | GTCTACGCCTGGAAAGCATGCCAA |
| PfimU5’ | GCATACCGTTGCCTGGCAACTGAT |
| PfimU3’FPmod | /5AmMC6/GTCTACGCCTGGAAAGCATGCCAA |
| **fimU EMSA** |  |
| PfimU5’ | GCATACCGTTGCCTGGCAACTGAT |
| PfimU3’ | GTCTACGCCTGGAAAGCATGCCAA |
| **Site directed mutagenesis** |  |
| fimU M1 5’ | GAACCGGGAGAAACG*TGAGA*TATGCA*TCACT*TCCGGATGATCATGGACC |
| fimU M1 3’ | GGTCCATGATCATCCGGA*AGTGA*TGCATA*TCTCA*CGTTTCTCCCGGTTC |
| fimU M2 5’ | GAACCGGGAGAAACG*TCAGC*TATGCA*GGACT*TCCGGATGATCATGGACC |
| fimU M2 3’ | GGTCCATGATCATCCGGA*AGTCC*TGCATA*GCTGA*CGTTTCTCCCGGTTC |
| algZ VBS1 5’ | GCGTGACG*GGCAA*AGGGCAACGATGA*TTACT*GGCCGGGAGGA |
| algZ VBS1 3’ | TCCTCCCGGCC*AGTAA*TCATCGTTGCCCT*TTGCC*CGTCACGC |
| algZ VBS2 5’ | CGGAA*GTATC*GCTGTT*CTGTG*GTTTGATTTGGCAGATGACATTTCGGTGC |
| algZ VBS2 3’ | GCACCGAAATGTCATCTGCCAAATCAAAC*CACAG*AACAGC*GATAC*TTCCG |
| algZ VBS3 5’ | CGGAACGAGCGCTGTTTCGCGGTTT*GATTT*GGCAGA*TGACA*TTTCGGTGC |
| algZ VBS3 3’ | GCACCGAAA*TGTCA*TCTGCC*AAATC*AAACCGCGAAACAGCGCTCGTTCCG |
| **algZ Start codon ablation** |  |
| algZ 5’ | TACAAAAAAGCAGGCTACCGGGCATGATGGTGTCGGCTTCCG |
| algZ 3’ | TACAAGAAAGCTGGGTGCGATCGCCAAGACGATACTGCTCAATG |
| algZ SOE 5’ | CACGGGCCTTGCGGAAACTCTGTCGTGATATCTCCGATTCAAGCCCGGCCGCTCGACC |
| algZ SOE 3’ | GGTCGAGCGGCCGGGCTTGAATCGGAGATATCACGACAGAGTTTCCGCAAGGCCCGTG |
| **algR deletion** |  |
| algR 5’ | TACAAAAAAGCAGGCTCACTATCAGCTGGGCGGAGAACTGAC |
| algR 3’ | TACAAGAAAGCTGGGTCGTAGCAGGCGATCGGCACCTGGCAAC |
| algR SOE 5’ | CCTGATTGTCGATGACGAACCTTGACGGCGGTCGGCGGTTCGCCAGCGAC |
| algR SOE 3’ | CGAACCGCCGACCGCCGTCAAGGTTCGTCATCGACAATCAGGACATTC |
